# Phenotypic plasticity in a novel set of EGFR tyrosine kinase inhibitor‐adapted non‐small cell lung cancer cell lines

**DOI:** 10.1002/2211-5463.70076

**Published:** 2025-06-26

**Authors:** Tharsagini V. Nanthaprakash, Campbell W. Gourlay, Ina Oehme, Michelle D. Garrett, Jindrich Cinatl, Mark N. Wass, Martin Michaelis

**Affiliations:** ^1^ School of Biosciences, Stacey Building University of Kent Canterbury UK; ^2^ Hopp Children's Cancer Center Heidelberg (KiTZ) Germany; ^3^ National Center for Tumor Diseases Heidelberg Germany; ^4^ Clinical Cooperation Unit Pediatric Oncology German Cancer Research Center (DKFZ) and German Cancer Consortium (DKTK) Heidelberg Germany; ^5^ Dr Petra Joh‐Research Institute Frankfurt am Main Germany

**Keywords:** acquired drug resistance, cell line, EGFR, EGFR tyrosine kinase inhibitor, non‐small cell lung cancer, plasticity

## Abstract

Here, we introduce novel sublines of the EGFR‐mutant non‐small cell lung cancer (NSCLC) cell lines HCC827 and HCC4006 adapted to the EGFR kinase inhibitors gefitinib (HCC827^r^GEFI^2μm^, HCC4006^r^GEFI^1μm^), erlotinib (HCC827^r^ERLO^2μm^, HCC4006^r^ERLO^1μm^) and afatinib (HCC827^r^AFA^50nm^, HCC4006^r^AFA^100nm^). All sublines displayed resistance to gefitinib, erlotinib, afatinib and the third‐generation EGFR kinase inhibitor osimertinib that overcomes T790M‐mediated resistance. HCC4006^r^ERLO^1μm^ displayed a spindle‐like morphology in agreement with previous findings that had detected epithelial–mesenchymal transition (EMT) in its precursor cell line HCC4006^r^ERLO^0.5μm^. EMT had also been reported for the HCC4006^r^GEFI^1μm^ precursor cell line HCC4006^r^GEFI^0.5μm^ and for HCC4006^r^AFA^100nm^, but the morphologies of HCC4006^r^GEFI^1μm^ or HCC4006^r^AFA^100nm^ did not support this, suggesting plasticity in EMT regulation during the drug adaptation process and in established resistant cell lines. Accordingly, HCC4006^r^ERLO^1μm^ displayed resistance to MEK and AKT inhibitors in contrast to its precursor HCC4006^r^ERLO^0.5μm^. We also detected metabolic plasticity, that is a temporary Warburg metabolism, in HCC4006 and HCC827^r^GEFI^2μm^. Response profiles to cytotoxic anticancer drugs, kinase inhibitors and HDAC inhibitors resulted in complex patterns that were specific for each individual subline, indicating individual resistance phenotypes. All resistant sublines remained sensitive or displayed collateral sensitivity to at least one of the investigated drugs. In conclusion, the comparison of EGFR kinase‐resistant NSCLC sublines with their precursor cell lines that had been previously characterised at a lower resistance level and metabolic investigations indicated phenotypic plasticity during the resistance formation process and in established cell lines. This plasticity may contribute to the well‐known variability in cell line phenotypes observed between different laboratories and in intra‐laboratory experiments.

## Graphical abstract

Phenotypic plasticity in a newly established set of EGFR inhibitor‐adapted NSCLC cell lines during adaptation and in established cell lines.

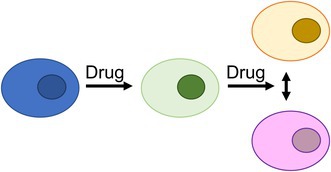

**Abbreviations**
EGFR: epidermal growth factor receptor
EMT: epithelial–mesenchymal transition
FBS: foetal bovine serum
HDAC: histone deacetylase
NSCLC: non‐small cell lung cancer

Lung cancer is responsible for the highest number of cancer‐related deaths, with 85% of cases being non‐small cell lung cancer (NSCLC) [1, 2, 3]. Many NSCLCs are driven by activating epithelial growth factor receptor (EGFR) mutations and are treated by first‐ (e.g. erlotinib, gefitinib), second‐ (e.g. afatinib) and/or third‐generation (e.g. osimertinib) EGFR tyrosine kinase inhibitors [1, 2, 3, 4, 5]. However, resistance formation after an initial therapy response is common, and new therapies are needed for NSCLC patients, whose tumours have stopped responding to EGFR tyrosine kinase inhibitor therapy [1, 2, 3, 4, 5].

Drug‐adapted (cancer) cell lines have been successfully used to identify clinically relevant resistance mechanisms since the 1970s [6, 7, 8, 9, 10, 11, 12, 13, 14]. Moreover, drug‐adapted cancer cell lines enable the detailed analysis of molecular acquired resistance mechanisms and the systematic testing of potential next‐line therapies [6, 8, 12, 13, 15, 16, 17].

Here, we introduce a novel set of NSCLC cell lines consisting of HCC827 and HCC4006 and their sublines adapted to gefitinib, erlotinib and afatinib. The results confirm previous experimental and clinical findings indicating that every resistance formation process follows a unique, unpredictable route. Moreover, they indicate that cancer cell lines are subject to phenotypic plasticity, both during the resistance formation process and as established cell lines.

## Materials and methods

### Cell culture

HCC4006 was purchased from ATCC (Manassas, VA, USA) and HCC827 from DSMZ (Braunschweig, Germany). Both cell lines are lung adenocarcinoma cell lines that harbour activating mutations (HCC4006: L747–E749 deletion, A750P; HCC827: E746–A750 deletion) in the EGFR tyrosine kinase domain.

The drug‐adapted sublines were established by continuous exposure to stepwise increasing drug concentrations as previously described [18] and derived from the Resistant Cancer Cell Line Collection (RCCL), (https://research.kent.ac.uk/industrial‐biotechnology‐centre/the‐resistant‐cancer‐cell‐line‐rccl‐collection/). Cell lines were selected based on their sensitivity to the respective drugs in the range of therapeutic plasma levels. The highest concentration that could be used for the continuous culturing of the cell lines was empirically selected as starting concentration.

All cell lines were cultured in Iscove's modified Dulbecco's medium (IMDM; Gibco™, Life Technologies, Cambridge, UK), supplemented with 10% (v/v) foetal bovine serum (FBS; Sigma‐Aldrich, Gillingham, UK), and 100 IU·mL^−1^ penicillin and 100 μg·mL^−1^ of streptomycin (Gibco™, Life Technologies), at 37 °C in a humidified 5% CO_2_ incubator. The media for resistant cell lines was additionally supplemented with the respective adaptation drug concentrations as specified by the cell line name. For example, HCC4006^r^AFA^100nm^ was maintained in 100 nm afatinib.

### Compounds

The following compounds were obtained from specified suppliers: Erlotinib, gefitinib, Afatinib, Osimertinib, Cisplatin, Paclitaxel (Selleckchem, Waltham Abbey, UK), Cabozantinib, Trametinib, Alpelisib, LY294002, Zosuquidar (MedChemExpress, Monmouth Junction, NJ, USA), AT13148 (Astex Pharmaceuticals, Cambridge, UK), Vincristine (Cayman Chemicals, Ann Arbor, MI, USA) and 2‐Deoxy‐d‐Glucose (Sigma‐Aldrich). All HDAC inhibitors were purchased from Selleckchem apart from apicidin and bufexamac, which were obtained from MedChemExpress and the selective HDAC8 inhibitor Compound 2, which was described elsewhere [19].

Most of the compounds were prepared and diluted using dimethyl sulfoxide (DMSO; Sigma‐Aldrich, Darmstadt, Germany) under sterile conditions and stored at −80 °C, except for cisplatin, which was dissolved in a 0.9% saline solution (0.9% (w/v)) and stored in dark tubes at room temperature.

### Cell imaging

The cell images were captured using bright field microscopy with a GXCAM‐U3‐5 industrial camera using different magnifications (Olympus CKX53 inverted microscope, Olympus Life Sciences, Southend‐on‐Sea, UK).

### Growth kinetics

The xCelligence real‐time cell analyser (RTCA) system was used to investigate the growth kinetics according to the manufacturer's instructions. Cells were grown in 16‐well microtiter plates (E‐Plate, ACEA Biosciences Inc., San Diego, CA, USA) in three replicates.

### Cell viability assays

If not stated differently, cell viability was determined by (3‐(4,5‐dimethylthiazol‐2‐yl)‐2,5‐diphenyltetrazolium bromide) MTT assay after 120‐h incubation as previously described [20]. Alternatively, the sulforhodamine B (SRB) assay was performed. Cells were fixed using 10% (w/v) trichloroacetic acid (TCA) and stained with 0.4% (w/v) SRB. Protein bound to SRB was then solubilised by adding 100 μL 10 mm Tris‐base per well (ThermoFisher Scientific, Waltham, MA, USA). Absorbance was determined using a Victor X4 Multilabel plate reader (PerkinElmerLife Sciences, High Wycombe, UK) at 490 nm. The percentage viability of drug‐treated cells was calculated relative to the untreated control. Then, the half‐maximal inhibitory concentrations (IC_50_) of the specific drug were determined using calcusyn software (Biosoft, Cambridge, UK).

### Determination of oxygen consumption rates

Oxygen consumption in cells was determined using Oxygraph‐2 k by Oroboros (Oroboros O2k, Oroboros Instruments GmbH, Innsbruck, Austria) following the manufacturer's instructions. 1 × 10^6^ cells·mL^−1^ cell suspensions were used per experiment. Oxygen consumption was monitored using the o2k software DatLab. After the system had reached an equilibrium (routine/basis respiration value), 2 μL of a (4 mg·mL^−1^) solution of the ATP synthase inhibitor oligomycin (Sigma‐Aldrich, Germany) was added to determine the oligomycin‐insensitive reduction in oxygen consumption (leak state). Then, 2 μL of 200 μm carbonyl cyanide 4‐(trifluoromethoxy)phenylhydrazone (FCCP) (Sigma‐Aldrich, Germany) solution was added to determine the maximum mitochondrial uncoupled respiration rate, referred to as the electron transport system value (ETS). Finally, 2 μL of 2 mm antimycin A (Sigma‐Aldrich, Germany) was added to measure the non‐mitochondrial oxygen consumption values (nMT). From the results, the ratios of mitochondrial routine respiration to ETS (Routine:ETS) and leak respiration to ETS (Leak:ETS) were calculated.

### Statistical analysis and data manipulation

Statistical analysis was performed using graphpad prism 10 (GraphPad Software, Inc., Boston, MA, USA). Two‐tailed *t*‐test used for single comparison assuming unequal variance. Multiple comparison was analysed using two‐way analysis of variance (ANOVA) followed by *post hoc* testing by Tukey's pairwise comparison/Dunnett's multiple comparison with 95% confidence interval.

## Results

### Resistance status, cell morphology and growth characteristics

**Fig. 1:**
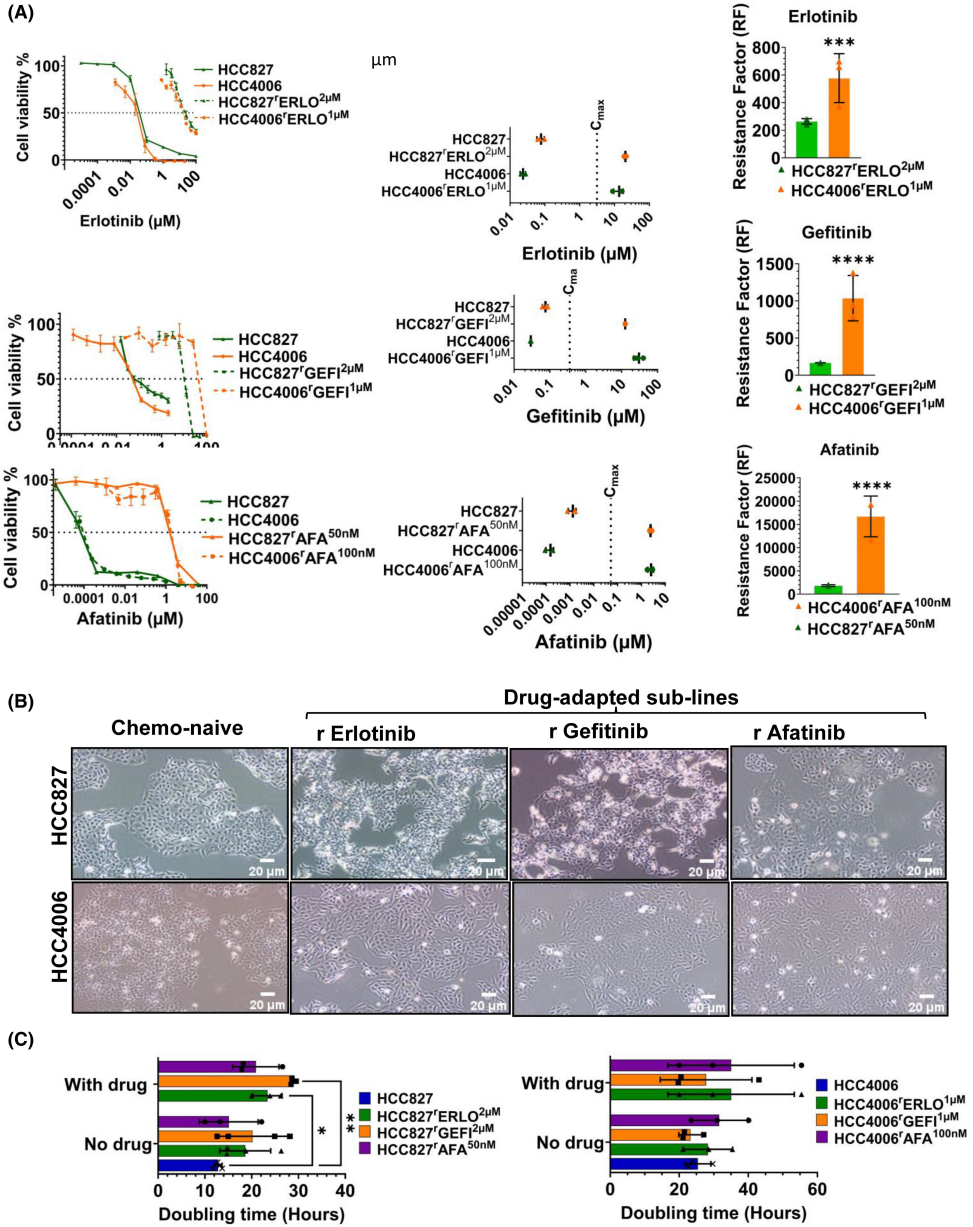
Basic characterisation of the EGFR tyrosine kinase inhibitor‐adapted HCC827 and HCC4006 sublines. (A) Dose–response curves, IC_50_ values and resistance factors (RF, IC_50_ resistant subline/IC_50_ respective parental cell line) demonstrating that the drug‐adapted sublines are resistant to the respective EGFR tyrosine kinase inhibitors relative to the respective parental cell lines. Cell viability was determined by MTT assay after a 120 h incubation period. IC_50_ values were calculated using the software calcusyn (Version 1.1, Biosof 1996). The data are from three independent biological repeats, mean ± SD. Differences were analysed for statistical significance (*P* < 0.05) by Student's *t*‐test. (B) Representative images of HCC827, HCC4006 and their EGFR receptor tyrosine kinase‐adapted sublines at 40× magnification (scale bar is 20 μm) (Olympus CKX53 inverted microscope, Olympus Life Sciences). (C) Doubling times of HCC827, HCC4006, and their EGFR receptor tyrosine kinase‐adapted sublines. In the resistant sublines, doubling times were determined in the absence and presence of the respective drugs of adaptation. The significance of differences between groups was analysed by two‐way analysis of variance (ANOVA) (*P* < 0.05), followed by *post hoc* test with Tukey's correction performed to compare individual cell lines compared to respective parental cells. The data are from three independent biological repeats, mean ± SD. *P < 0.05, **P < 0.01. IC_50_ – half maximal inhibitory concentrations.

**Fig. 2:**
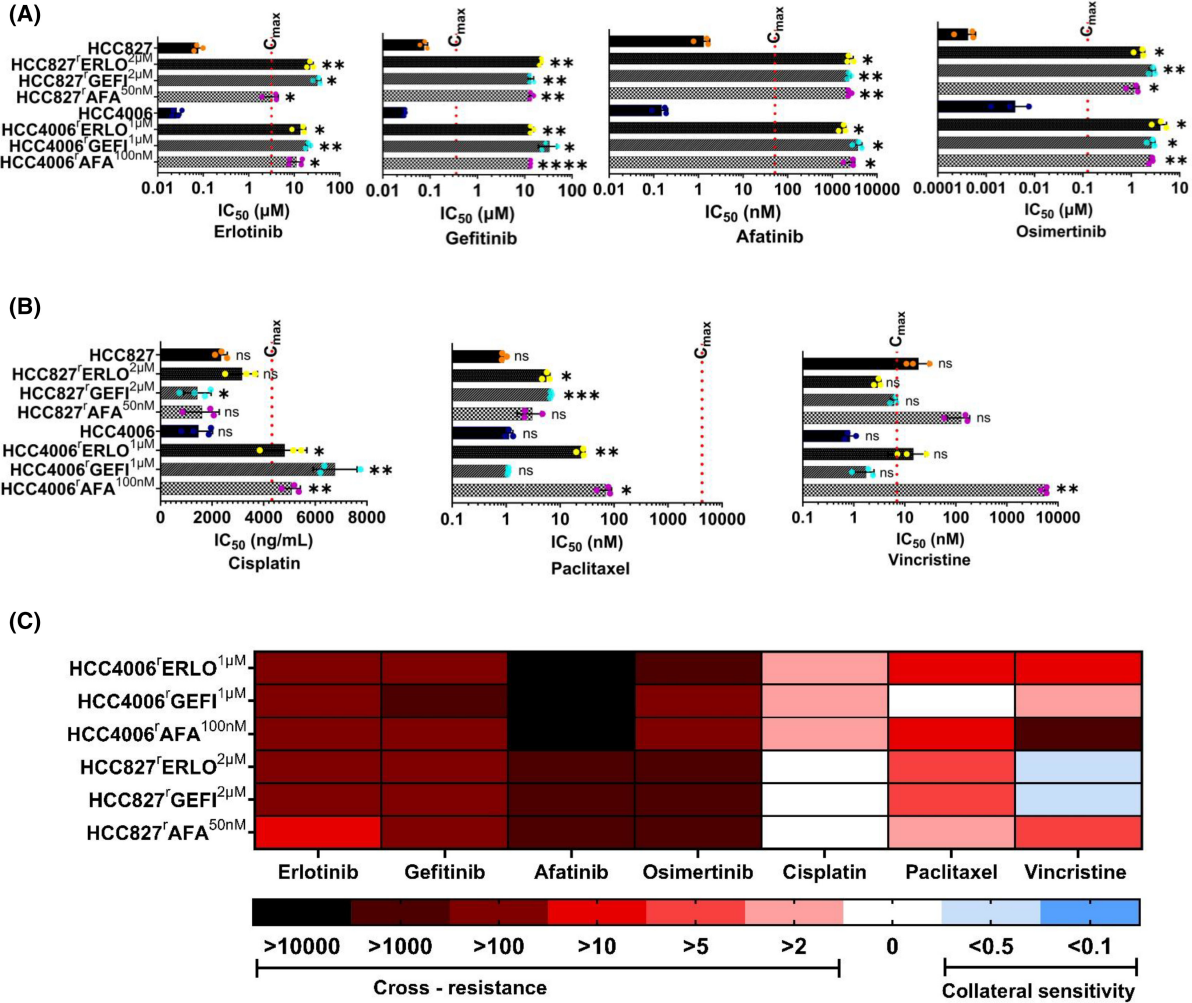
Cell line sensitivity profiles to selected anticancer drugs. Cell viability was determined by MTT assay after a 120 h incubation period. IC_50_ values were calculated using the software calcusyn (version 1.1, Biosof 1996). The data are from three independent biological repeats, mean ± SD. Red dotted line represents the clinical maximum plasma concentrations (*C*_max_) of respective drugs. Differences were analysed for statistical significance (*P* < 0.05) by Student's *t*‐test. *P < 0.05, **P < 0.01, ***P < 0.001, ****P < 0.0001. (A) Sensitivity of HCC827, HCC4006, and their EGFR receptor tyrosine kinase‐adapted sublines to different EGFR tyrosine kinase inhibitors. Dose–response curves are presented in Fig. S1. (B) Sensitivity of HCC827, HCC4006 and their EGFR receptor tyrosine kinase‐adapted sublines to cisplatin, paclitaxel, and vincristine. Dose–response curves are presented in Fig. S2. (C) Overview heatmap providing the response profiles of the cell lines to the investigated drugs based on the resistance factors (RF, IC_50_ resistant subline/IC_50_ respective parental cell line). Resistance (red) was defined as an RF > 2, comparable sensitivity (white) as RF ≤ 2 and ≥ 0.5, and increased sensitivity/collateral vulnerability (blue) as RF < 0.5. IC_50_ – half maximal inhibitory concentrations.

The results of the initial characterisation of the project cell lines for sensitivity to the respective drugs of adaptation, cell morphology and doubling times are presented in Fig. 1. All EGFR tyrosine kinase inhibitor‐adapted sublines displayed high levels of resistance to the respective inhibitors (Fig. 1A). The parental cell lines were sensitive to clinically achievable therapeutic plasma concentrations (*C*_max_) of the respective EGFR tyrosine kinase inhibitors [21], while the IC_50_ values for all the resistant sublines were above the *C*_max_ values (Figs 1A and 2A).

Representative images of the cell lines are provided in Fig. 1B. HCC827 and HCC827^r^AFA^50nm^ displayed an enhanced adhesion to the cell flask surface compared to HCC827^r^ERLO^2μm^ and HCC827^r^GEFI^2μm^ (and to HCC4006 and its sublines). A trypsin concentration of 0.12% (w/v) was required to detach HCC827 and HCC827^r^AFA^50nm^ during passaging, while a trypsin concentration of 0.05% (w/v) was used for the remaining HCC827 sublines. Moreover, HCC827 and HCC827^r^AFA^50nm^ grew in monolayers, while HCC827^r^ERLO^2μm^ and HCC827^r^GEFI^2μm^ formed multilayers.

Among HCC4006 and its sublines, HCC4006^r^ERLO^1μm^ displayed a more spindle‐like morphology, while HCC4006^r^GEFI^1μm^ and HCC4006^r^AFA^100nm^ had a wider diameter than HCC4006 (Fig. 1B). Moreover, HCC4006 formed multilayers in contrast to its sublines, which all grew as monolayers.

The doubling times of the EGFR tyrosine kinase‐adapted HCC827 and HCC4006 sublines did not significantly differ from the respective parental cell lines (Fig. 1C, Table S1). The addition of the respective drugs of adaptation only affected the doubling times of two of the sublines: The doubling time of HCC827^r^GEFI^2μm^ was 28.8 ± 0.5 h in the presence of gefitinib 2 μm and 20.2 ± 7.3 h in the absence of drug. Moreover, the doubling time of HCC827^r^ERLO^2μm^ was 23.4 ± 2.8 h in the presence of erlotinib 2 μm and 18.6 ± 4.3 h in the absence of drug (Fig. 1C, Table S1).

### EGFR tyrosine kinase inhibitor and cytotoxic drug response profiles

Next, we determined the response profiles of the project cell lines to the clinically approved EGFR tyrosine kinase inhibitors erlotinib, gefitinib, afatinib and osimertinib and the cytotoxic anticancer drugs cisplatin, paclitaxel and vincristine. The results are provided in Fig. 2. Erlotinib and gefitinib are first‐generation EGFR kinase inhibitors. Afatinib is a second‐generation EGFR kinase inhibitor. Osimertinib is a third‐generation EGFR kinase inhibitor designed to overcome resistance to first‐ and second‐generation EGFR kinase inhibitors that is mediated by T790M EGFR mutations [2, 5].

All resistant sublines displayed pronounced resistance against all four EGFR inhibitors (Fig. 2A, Fig. S1, Table S2), indicating that EGFR kinase inhibitor resistance is not driven by T790M mutations in the project cell lines. The resistance factors (IC_50_ resistant subline/IC_50_ respective parental cell line) ranged from 42.2 (HCC827^r^Afa^50nm^, erlotinib) to 24815.1 (HCC4006^r^Gefi^1μm^, afatinib) (Table S2).

Cytotoxic chemotherapy remains an option for the treatment of EGFR‐mutant NSCLC after resistance formation to EGFR kinase inhibitors [22, 23, 24, 25]. Platinum‐based therapies are among the most commonly used cytotoxic anticancer drugs used at different stages of the treatment of EGFR‐mutant NSCLC [5, 22, 24, 25, 26, 27]. Moreover, taxanes and vinca alkaloids belong to the chemotherapeutic drug classes that are still investigated for the treatment of EGFR‐mutant NSCLC [5, 23, 27, 28].

Here, we investigated the response of the cell lines from our panel to the platinum drug cisplatin, the taxane paclitaxel (stabilising tubulin‐binding agent) and the vinca alkaloid vincristine (destabilising tubulin‐binding agent) (Fig. 2B, Fig. S2, Table S3).

When analysing the resistance profiles, we considered resistance factors (IC_50_ resistant subline/IC_50_ respective parental cell line) ≥ 2 as cross‐resistance, resistance factors < 2 and > 0.5 as similar sensitivity, and resistance factors ≤ 0.5 as increased sensitivity/collateral vulnerability. The drug response profiles revealed a substantial level of heterogeneity among the EGFR kinase inhibitor‐resistant NSCLC sublines (Fig. 2C).

All HCC827 sublines displayed similar cisplatin sensitivity as HCC827 with resistance factors ranging from 0.61 (HCC827^r^GEFI^2μm^) to 1.35 (HCC827^r^ERLO^2μm^) (Fig. 2B, Table S3A). In contrast, all HCC4006 sublines were cross‐resistant to cisplatin with resistance factors ranging from 3.26 (HCC4006^r^ERLO^1μm^) to 4.58 (HCC4006^r^GEFI^1μm^) (Fig. 2B, Table S3B).

All EGFR kinase‐resistant sublines, except for HCC4006^r^GEFI^1μm^ cells (resistance factor 0.96), displayed cross‐resistance to paclitaxel (Fig. 2B, Table S3B). However, the resistance factors varied considerably among the cross‐resistant sublines ranging from 3.38 (HCC827^r^AFA^50nm^) to 61.5 (HCC4006^r^AFA^100nm^).

The vincristine response profiles were associated with the highest level of variability (Fig. 2B, Table S3). All HCC4006 sublines and HCC827^r^AFA^50nm^ cells displayed cross‐resistance with resistance factors ranging from 2.06 (HCC4006^r^GEFI^1μm^) to 5451 (HCC4006^r^AFA^100nm^), while HCC827^r^ERLO^2μm^ cells (resistance factor 0.16) and HCC827^r^GEFI^2μm^ cells (resistance factor 0.33) displayed collateral vulnerability.

### Kinase inhibitor response profiles

**Fig. 3:**
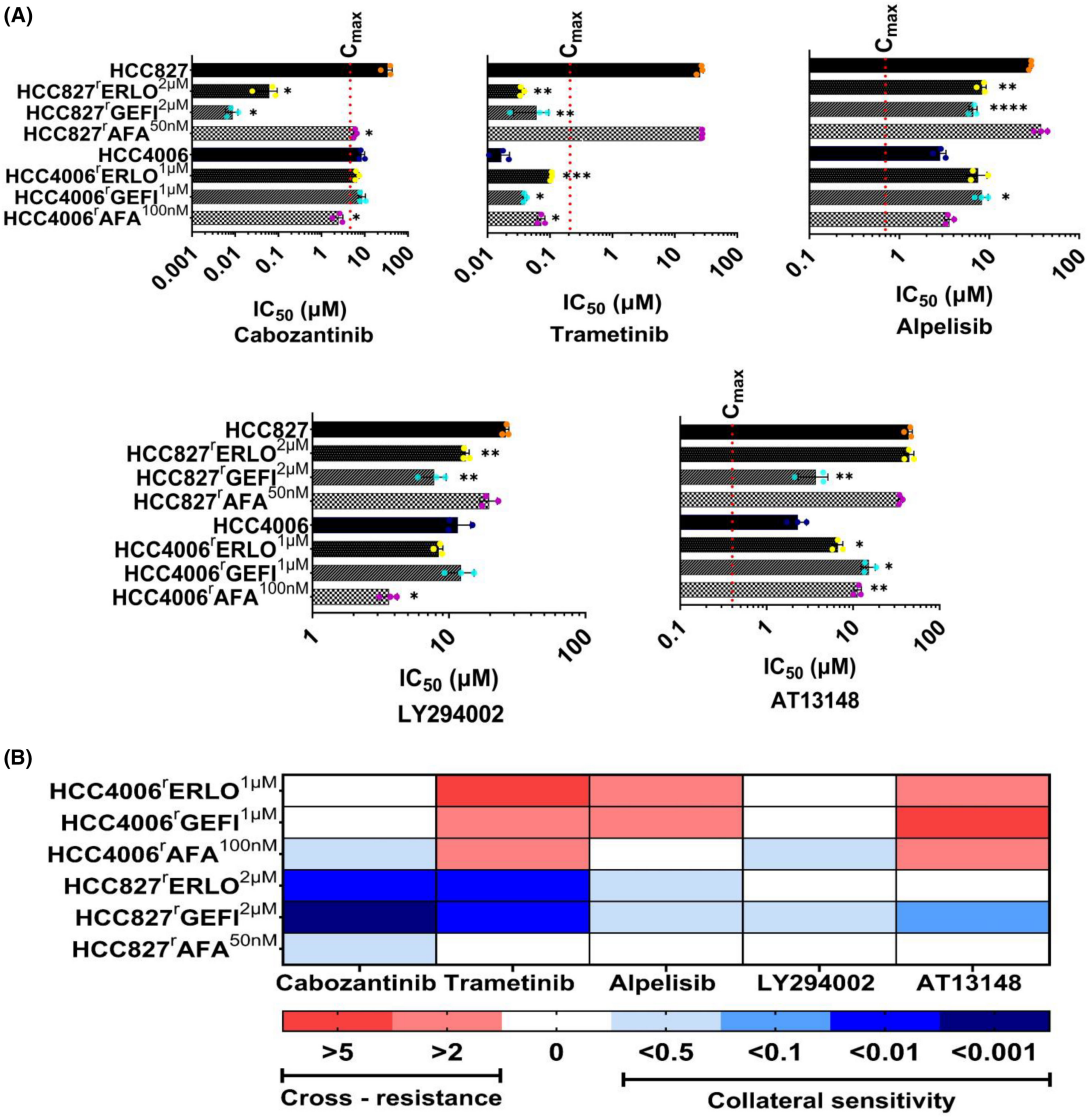
Cell line sensitivity profiles to selected kinase inhibitors. Cell viability was determined by MTT assay after a 120 h incubation period. IC_50_ values were calculated using the software calcusyn (Version 1.1, Biosof 1996). (A) Sensitivity of HCC827, HCC4006 and their EGFR receptor tyrosine kinase‐adapted sublines to different kinase inhibitors. Values represents mean ± SD of three independent experiments. Dose–response curves are presented in Fig. S3. Red dotted line represents the clinical maximum plasma concentrations (*C*_max_) of respective the drugs. Differences were analysed for statistical significance (*P* < 0.05) by Student's *t*‐test. *P < 0.05, **P < 0.01, ***P < 0.001, ****P < 0.0001. (B) Overview heatmap providing the response profiles of the cell lines to the investigated drugs based on the resistance factors (RF, IC_50_ resistant subline/IC_50_ respective parental cell line). Resistance (red) was defined as an RF > 2, comparable sensitivity (white) as RF ≤ 2 and ≥ 0.5, and increased sensitivity/collateral vulnerability (blue) as RF < 0.5. IC_50_ – half maximal inhibitory concentrations.

In EGFR‐mutant NSCLC, oncogenic signalling by constitutive active EGFR is mediated via the RAS/RAF/MEK/ERK (MAPK) and/or PI3K/AKT signalling pathways. In agreement, common EGFR kinase inhibitor resistance mechanisms include the reinstatement of MAPK and PI3K/AKT signalling by mechanisms including MET amplification [2, 5, 25, 29]. Hence, we next investigated the effects of MET (cabozantinib), MEK (trametinib) *post‐hoc* and PI3K (alpelisib, LY294002) inhibitors on the project cell lines. We also included AT13148, an inhibitor of AGC kinases including AKT, ROCK1/2 and p70S6K [30], into our analysis. The results are shown in Fig. 3.

Overall, the EGFR tyrosine kinase‐adapted NSCLC sublines displayed complex response patterns to the investigated kinase inhibitors (Fig. 3, Fig. S3, Table S4). The HCC827 sublines showed similar or increased sensitivity to all kinase inhibitors relative to HCC827. Among the HCC827 sublines, the kinase inhibitor response profiles were more similar between HCC827^r^ERLO^2μm^ (collateral vulnerability to all tested kinase inhibitors except AT13148) and HCC827^r^GEFI^2μm^ (collateral vulnerability to all tested kinase inhibitors) compared to HCC827^r^AFA^50nm^ (collateral vulnerability only to cabozantinib) (Fig. 3, Table S4).

In contrast to the HCC827 sublines, the HCC4006 sublines were cross‐resistant to a number of kinase inhibitors (Fig. 3, Table S4). Again, the erlotinib‐ and gefitinib‐adapted sublines displayed a higher level of similarity versus the afatinib‐resistant subline. HCC4006^r^ERLO^1μm^ and HCC4006^r^GEFI^1μm^ were both cross‐resistant to trametinib, alpelisib, and AT13148 and similarly sensitive as HCC4006 to cabozantinib and LY294002. HCC4006^r^AFA^100nm^ showed collateral vulnerability to cabozantinib and LY294002, cross‐resistance to trametinib and AT13148, and similar sensitivity as HCC4006 to alpelisib (Fig. 3, Table S4B). It remains unclear to which extent the differences in the kinase inhibitor response profiles between the HCC827‐ and HCC4006‐sublines are the consequence of the different cellular backgrounds and/or of chance events during the adaptation process.

It is difficult to draw conclusions from the kinase inhibitor profiles on whether the resistance formation process may have resulted in a dependence on certain signalling pathways in the sublines. This is most obviously demonstrated by the inconsistent responses of the project cell lines to alpelisib, LY294002 and AT13148 that all target PI3K/AKT signalling (Fig. 3, Table S4). Only HCC827^r^GEFI^2μm^ displayed a consistent response (collateral vulnerability) to all three compounds, which might indicate an increased dependence on PI3K/AKT signalling. However, HCC827^r^GEFI^2μm^ also showed increased sensitivity to the MET inhibitor cabozantinib and the MEK inhibitor trametinib (Fig. 3, Table S4A), which suggests multiple resistance mechanisms.

**Fig. 4:**
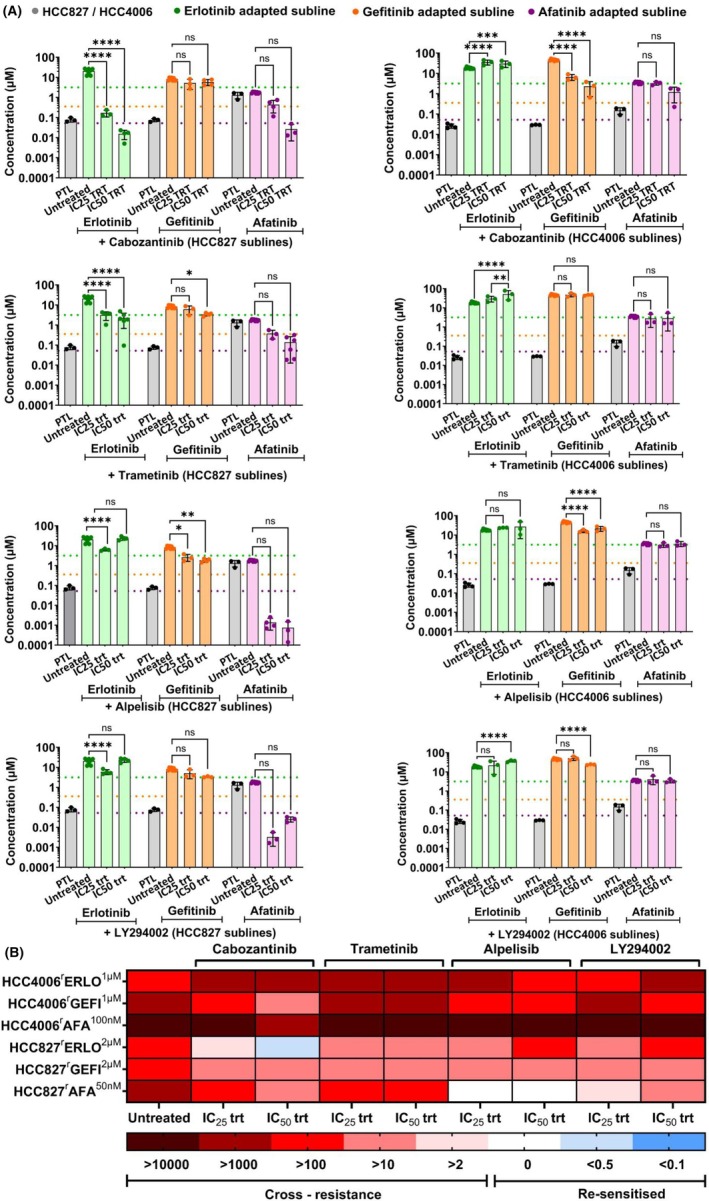
Effect of kinase inhibitors on the sensitivity of EGFR tyrosine kinase inhibitor‐adapted cell lines to the respective drugs of adaptation. Cell viability was determined by MTT assay after a 120 h incubation period. IC_50_ values were calculated using the software calcusyn (version 1.1, Biosof 1996). (A) Effect of the kinase inhibitors IC_25_ and IC_50_ concentrations on the sensitivity of the EGFR tyrosine kinase inhibitor‐resistant sublines to the respective drugs of adaptation. Values represent mean ± SD of three independent experiments (*n* = 4 for IC_25_ of alpelisib and LY294002 when treated along with erlotinib, *n* = 5 for trametinib when treated along with IC_25_ of trametinib). Dose–response curves are presented in Figs S4–S6. Differences were analysed for statistical significance (*P* < 0.05) by two‐way ANOVA with *post hoc* Tukey's multiple comparison test. *P < 0.05, **P < 0.01, ***P < 0.001, ****P < 0.0001. (B) Overview heatmap providing the response profiles of the cell lines to the investigated drugs based on the relative resistance (IC_50_ without kinase inhibitor/IC_50_ with kinase inhibitor). Increased resistance (red) was defined as > 2, comparable sensitivity (white) as ≤ 2 and ≥ 0.5, and increased sensitivity/collateral vulnerability (blue) as RF < 0.5. IC_50_ – half maximal inhibitory concentrations, IC_25_ – concentration that inhibit cell viability by 25%.

Next, we treated the project cell lines with the EGFR kinase inhibitors in the presence of IC_25_ and IC_50_ concentrations of the kinase inhibitors (Table S5) to see whether kinase inhibitors may re‐sensitise the sublines to the respective EGFR kinase inhibitors (Fig. 4, Figs S4–S6, Table S6). In most cell lines, the kinase inhibitors had no or only modest (cabozantinib/HCC4006^r^GEFI^1μm^, trametinib/HCC827^r^ERLO^2μm^ trametinib/HCC827^r^AFA^50nm^) effects on the efficacy of the EGFR kinase inhibitors to which the sublines had been adapted to (Fig. 4, Table S6).

Cabozantinib exhibited slightly more pronounced effects on the afatinib activity in HCC827^r^AFA^50nm^ and LY294002 on the afatinib activity in HCC827^r^AFA^50nm^. Re‐sensitisation to the level of the parental cell line was only achieved by cabozantinib in HCC827^r^ERLO^2μm^ and by alpelisib in HCC827^r^AFA^50nm^ (Fig. 4, Table S6). Again, it is difficult to draw meaningful mechanistic conclusions from these results, not least because the PI3K inhibitors alpelisib and LY294002 differed in their activities.

Off‐target resistance mechanisms that interfere with compound transport into or out of cancer cells can mediate resistance on their own or in combination with on‐target resistance mechanisms that directly affect the drug target and the related signalling pathways [31]. In this context, erlotinib, gefitinib and afatinib are known to be substrates of the ATP‐binding cassette transporter ABCB1 (also known as P‐glycoprotein or MDR1) [32], a major transporter involved in drug resistance in cancer [31]. ABCB1 can also mediate acquired EGFR tyrosine kinase inhibitor resistance [33, 34]. However, using the ABCB1 substrate vincristine [35] in combination with the specific third‐generation ABCB1 inhibitor zosuquidar [35] as previously described [20, 36], indicated that only HCC4006^r^ERLO^1μm^ displayed an ABCB1‐mediated resistance phenotype (Fig. S7). This indicates that ABCB1‐mediated effects have limited impact on the tyrosine kinase inhibitor response profiles in our panel of EGFR tyrosine kinase‐adapted NSCLC sublines.

Notably, other ABC transporters may also be involved in EGFR kinase inhibitor resistance [32, 35]. Moreover, kinase inhibitors including those that we used in our study are known to display complex interactions with a range of kinases in addition to the desired ones [37, 38, 39]. Hence, we can conclude that each of the EGFR kinase inhibitor‐adapted cell lines has developed a unique phenotype, but future research will have to elucidate the exact resistance mechanisms in each cell line in detail.

### Role of histone deacetylases (HDACs) in EGFR kinase resistance

Changes in histone deacetylase (HDAC) regulation contribute to cancer formation and progression and can also contribute to cancer cell resistance to anticancer drugs, including EGFR kinase inhibitors [40, 41, 42]. The role of HDACs in cancer has resulted in the design of numerous HDAC inhibitors [43]. While the first compounds typically were pan‐HDAC inhibitors, there has recently been more focus on the development of isotype‐specific HDAC inhibitors [11, 43, 44, 45, 46].

**Fig. 5:**
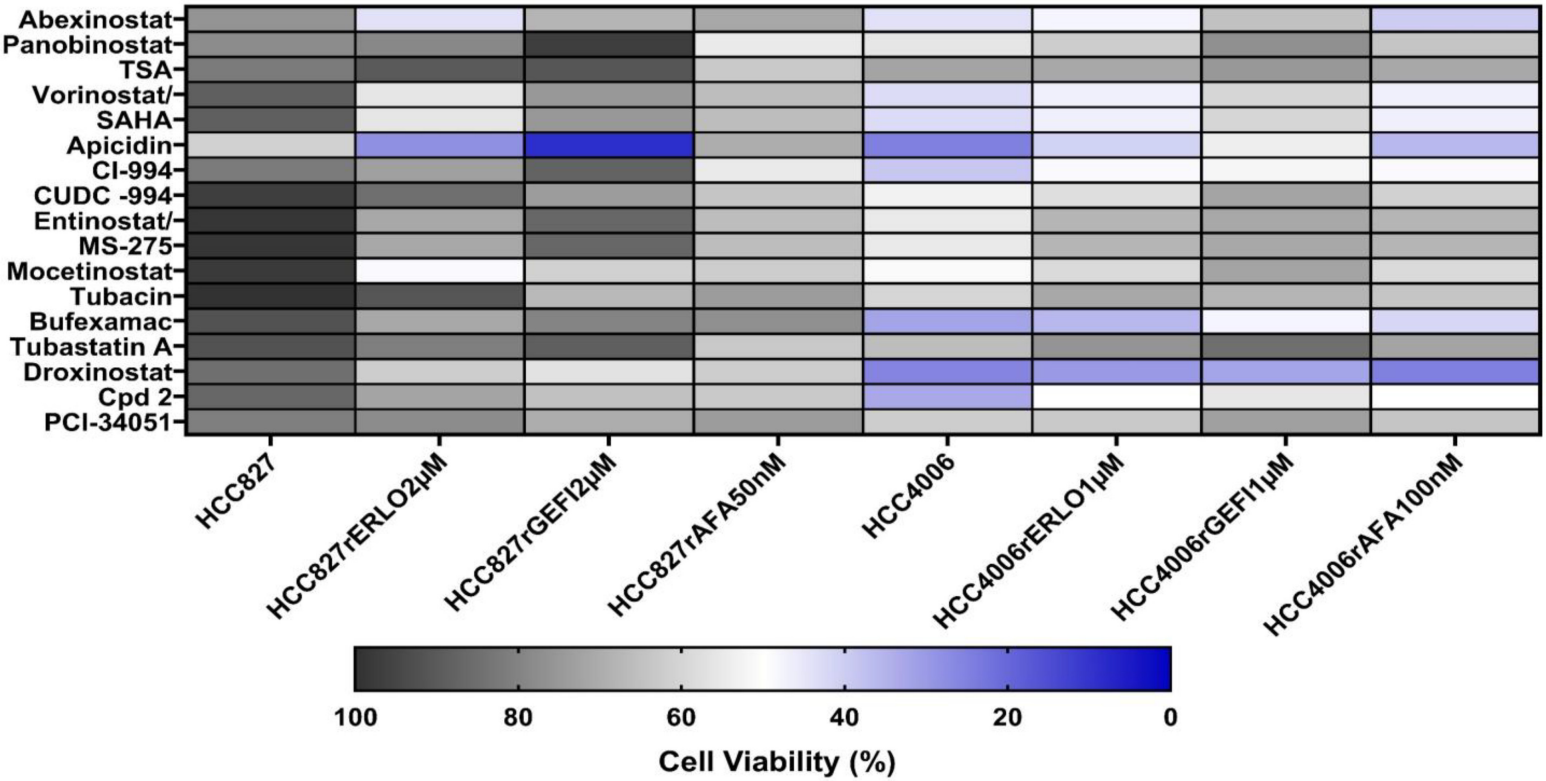
Effects of fixed HDAC inhibitor concentrations on cell viability. Cell viability was determined MTT assay after 120 h of incubation. The rationale behind selecting the indicated concentrations is presented in Table S7. The numerical data are presented in Table S8.

Figure 5 reports on the characterisation of our cell line panel using a set of 15 HDAC inhibitors, each at a concentration selected to interfere specifically with one or a limited number of HDACs (Table S7). HCC827 displayed a generally lower sensitivity to HDAC inhibitors than HCC4006 (Fig. 5, Table S8). Some resistant sublines displayed an increased sensitivity to certain HDAC inhibitors, in particular HCC827^r^GEFI^2μm^ to apicidin (Fig. 5, Table S8). However, other HDAC inhibitors that like apicidin inhibit HDAC1, HDAC2 and HDAC3 did not exert comparable effects. Hence, no general mechanistic insights can be drawn from the HDAC inhibitor data. Nevertheless, the findings show that resistance formation to EGFR tyrosine kinase inhibitors can be associated with increased sensitivity to certain HDAC inhibitors. Further research will be needed to investigate the role of HDACs in acquired EGFR kinase inhibitor resistance in more detail. Nevertheless, the findings further confirm that each EGFR kinase‐resistant subline has developed an individual phenotype.

### Role of the individual cell line background in EGFR kinase resistance formation

**Fig. 6:**
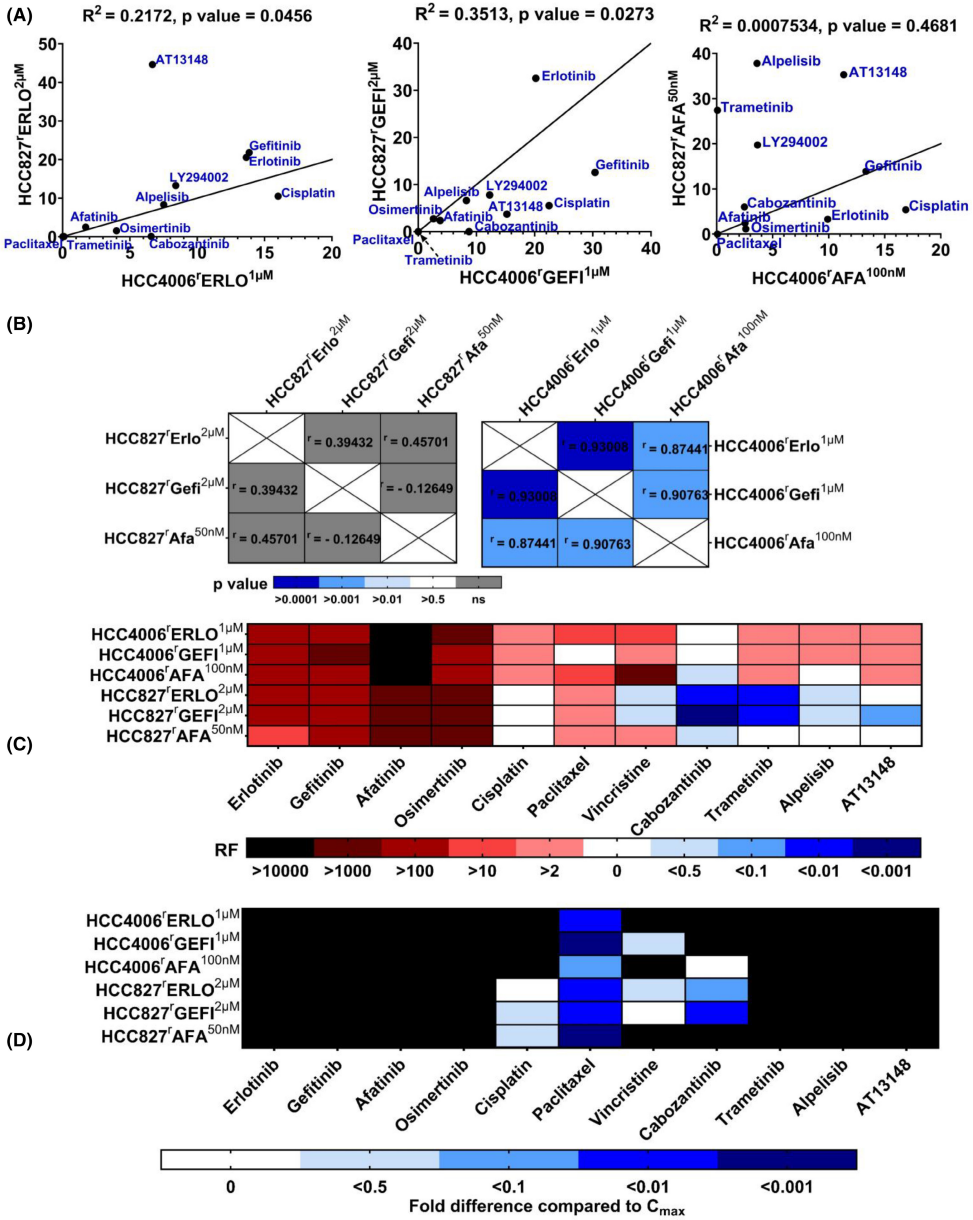
Correlation of the drug response patterns among the EGFR tyrosine kinase‐adapted sublines. (A) Correlation of the drug response profiles among the sublines adapted to the same drug. The straight line represents the best fit. (B) Correlation of the drug response profiles among the sublines of the same parental cell lines. (C) The heatmap summarising the drug sensitivity profiles of the EGFR tyrosine kinase inhibitor‐adapted sublines relative to the respective parental cell lines expressed as resistance factors (RF, IC_50_ resistant subline/IC_50_ respective parental cell line). (D) Heatmap summarising cell line sensitivity to therapeutic plasma concentrations (*C*_max_) of the indicated drugs. Black indicates IC_50_ values higher than the *C*_max_. IC_50_ – half maximal inhibitory concentrations.

The determination of drug response profiles indicated complex, individual phenotypes among the EGFR kinase inhibitor sublines. The correlation of the drug response profiles among the sublines adapted to the same drug demonstrated significant but not very pronounced correlations among the erlotinib‐ and gefitinib‐resistant sublines and no significant correlation among the afatinib‐adapted sublines (Fig. 6A). The correlation of the drug response profiles among the sublines of the same parental cell lines demonstrated significant correlations among the HCC4006 sublines but not among the HCC827 sublines (Fig. 6B). Based on these findings, it remains unclear if, and if yes, to what extent, there are similarities between sublines adapted to the same drug and to what extent the cellular backgrounds contribute to the resistance phenotypes.

### Cross‐resistance profiles in the context of clinically achievable therapeutic concentrations

The complexity of the phenotypes of the EGFR kinase inhibitor‐adapted sublines can also be illustrated by an overarching heatmap reflecting the response to EGFR kinase inhibitors, additional kinase inhibitors, and cytotoxic anticancer drugs (Fig. 6C). Moreover, when we considered the drug activities in the context of clinically achievable plasma concentrations (*C*_max_) [21], this interestingly resulted in different sensitivity patterns (Fig. 6D). This indicates that drug response correlations derived from model systems are primarily insightful with regard to mechanistic considerations. For translational approaches, the clinically achievable plasma concentrations also need to be taken into account.

### Reversible shift in cell line oxygen consumption

Cancer cells may undergo a metabolic shift resulting in ATP production under normoxic conditions via glycolysis (instead of oxidative phosphorylation in the mitochondria), a phenomenon referred to as ‘aerobic glycolysis’ and/or ‘Warburg effect’ [47]. Such metabolic changes can also be associated with reduced cancer cell sensitivity to anticancer drugs, including EGFR tyrosine kinase inhibitors [47, 48].

The MTT assay was used for the determination of drug effects in this study, which measures oxidative phosphorylation in the mitochondria as a surrogate for cell viability [20, 49, 50]. Hence, the MTT assay is not suited for cells displaying a Warburg metabolism. During the course of the project, we occasionally observed that the signals from the cell untreated controls in HCC4006 and HCC827^r^GEFI^2μm^ did not significantly differ from the background signals of the cell culture media only controls, although microscopic inspection revealed viable, confluent cell layers in the respective former.

**Fig. 7:**
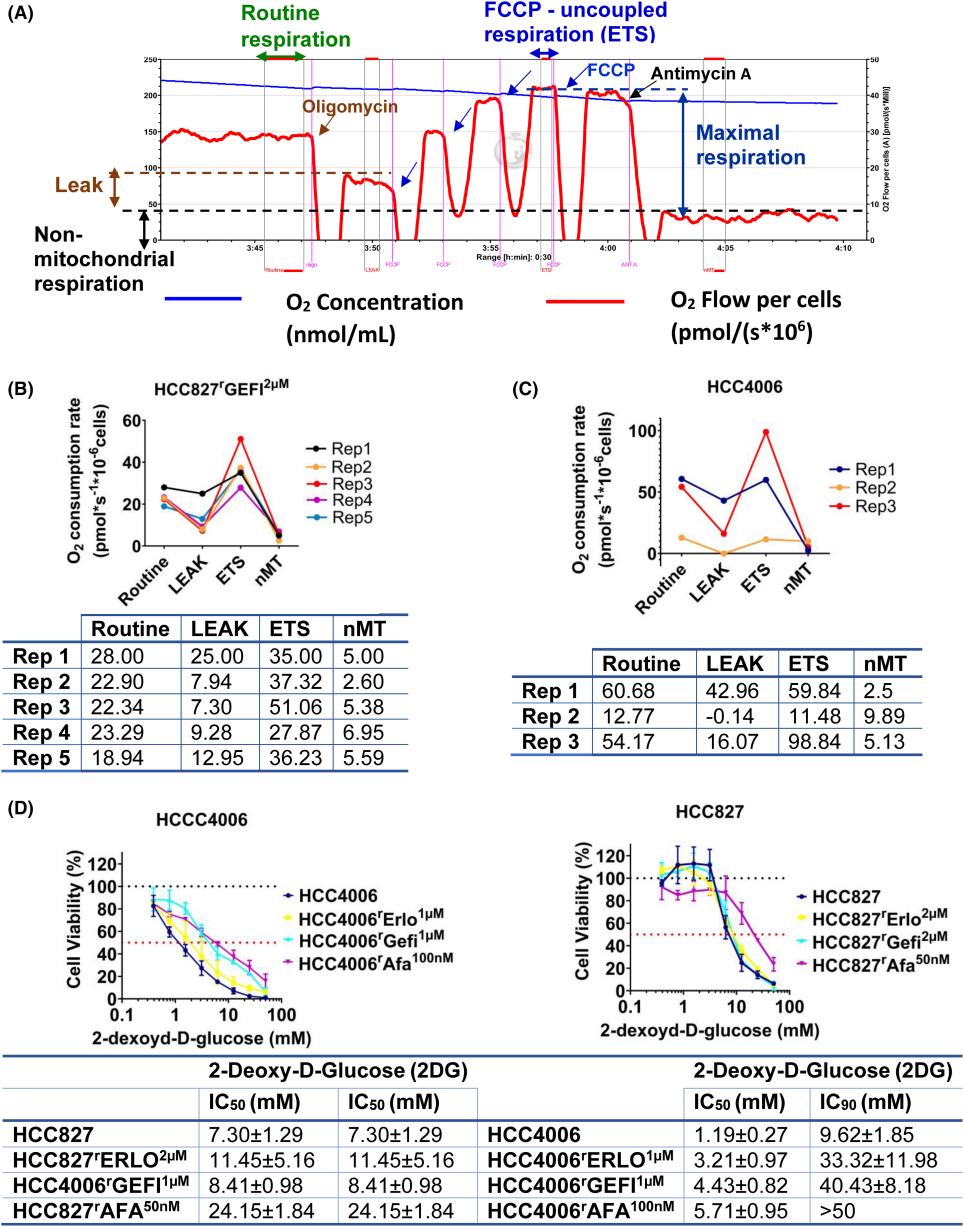
Measurement of oxygen consumption. Oxygen consumption was measured using an Oxygraph‐2 k respirometer. (A) Representative high‐resolution respirometry (HRR) profile of HCC827. Routine respiration is shown in green. Addition of the ATP synthase inhibitor oligomycin then causes a reduction in oxygen consumption (LEAK). FCCP (carbonyl cyanide 4‐(trifluoromethoxy)phenylhydrazone) causes mitochondrial membrane permeabilisation, resulting in maximum respiration/ oxygen consumption (uncoupling, ETS = electron transfer system capacity). Complex III inhibitor antimycin A addition results in respiration suppression, indicating non‐mitochondrial oxygen consumption. (B) Respirometry results of HCC827^r^GEFI^2μm^ cells from five independent experiments. (C) Respirometry results of HCC4006 cells from three independent experiments. (D) Effect of 2‐Deoxy‐d‐Glucose (2DG) on cell line viability as indicated by MTT assay after 120 h incubation. IC_50_ values were calculated using the software calcusyn (version 1.1, Biosof 1996). The data are from three independent biological repeats, means ± SD. IC_50_ – half maximal inhibitory concentrations.

To investigate this phenomenon, we determined cellular oxygen consumption in the project cell lines. Moreover, the sensitivity of the project cell lines was tested against 2‐Deoxy‐d‐Glucose (2DG), a competitive inhibitor of glycolysis with activity against cancer cells displaying a Warburg phenotype [51]. The respective results are provided in Fig. 7.

A standard respiration profile is provided in Fig. 7A. Cells are monitored until a stable routine respiration level has been established. In cells with intact oxidative phosphorylation, the addition of the ATP synthase inhibitor oligomycin then causes a reduction in oxygen consumption (leak state). Next, the addition of FCCP (carbonyl cyanide 4‐(trifluoromethoxy)phenylhydrazone), a protonophore that causes mitochondrial membrane permeabilisation, results in maximum respiration/oxygen consumption (uncoupling, ETS = electron transfer system capacity). Finally, the complex III inhibitor antimycin A is added, resulting in the complete suppression of respiration, indicating non‐mitochondrial oxygen consumption (Fig. 7A). Small or no differences in oxygen consumption during the different assay steps indicate a lack of oxidative phosphorylation in the mitochondria.

Indeed, HCC4006 (Fig. 7B) and HCC827^r^GEFI^2μM^ (Fig. 7C) displayed temporary shifts towards a Warburg metabolism. However, the changes in metabolism occurred only sporadically and were unpredictable, which made it unfeasible to investigate this phenomenon systematically during this project. Nevertheless, the metabolism of these two cell lines seemed to fluctuate occasionally between oxidative phosphorylation and aerobic glycolysis. None of the other cell lines displayed similar shifts in their metabolism (Fig. S8).

2DG treatment resulted in complex results. HCC4006 and its sublines were generally more sensitive to HCC827 and its sublines, and HCC4006 was indeed the most 2DG‐sensitive cell line of the panel (Fig. 7D). However, the 2DG sensitivity of HCC827^r^GEFI^2μm^ was substantially lower and similar to that of HCC827 (Fig. 7D). Notably, we were not able to measure the 2DG sensitivity of HCC4006 and HCC827^r^GEFI^2μm^ when they were in an acute aerobic glycolysis metabolic phase. Further research will have to elucidate this metabolic plasticity of these cell lines further.

## Discussion

Here, we introduce a novel panel of NSCLC cell lines consisting of the EGFR‐mutant cell lines HCC827 and HCC4006 and their sublines adapted to the EGFR tyrosine kinase inhibitors gefitinib (HCC827^r^GEFI^2μm^, HCC4006^r^GEFI^1μm^), erlotinib (HCC827^r^ERLO^2μm^, HCC4006^r^ERLO^1μm^) and afatinib (HCC827^r^AFA^50nm^, HCC4006^r^AFA^100nm^). Notably, all resistant sublines displayed resistance to gefitinib, erlotinib and afatinib and also to the third‐generation EGFR kinase inhibitor osimertinib, which was developed to overcome resistance mediated by T790M EGFR mutations [2, 5]. Hence, these findings indicate that the resistance in the EGFR kinase inhibitor‐adapted sublines is not mediated by T790M mutations, but rather by EGFR‐independent mechanisms that mediate resistance to first‐, second‐, and third‐generation EGFR tyrosine kinase inhibitors [2, 5, 52]. Hence, our results also indicate that while acquired EGFR kinase inhibitor resistance may be delayed in some cases by designing further generations of EGFR kinase inhibitors that target additional EGFR resistance mutations [52], resistance is likely to emerge eventually by EGFR‐independent processes. Accordingly, the combination of amivantamab, a bispecific antibody targeting EGFR and MET, with the third‐generation EGFR kinase inhibitor lazertinib was found to be superior to lazertinib or osimertinib alone [53].

Resistance formation resulted in morphological changes in some of the sublines. Most notably, HCC4006^r^ERLO^1μm^ displayed a more spindle‐like morphology compared to its parental cell line HCC4006. This is in agreement with findings that had suggested that the HCC4006^r^ERLO^1μm^ precursor cell line HCC4006^r^ERLO^0.5μm^ displayed an epithelial–mesenchymal transition (EMT) phenotype [54]. However, the same study had also reported EMT for the HCC4006^r^GEFI^1μm^ precursor cell line HCC4006^r^GEFI^0.5μm^ and for HCC4006^r^AFA^100nm^ [54], but we did not find any morphological indications of this in HCC4006^r^GEFI^1μm^ or HCC4006^r^AFA^100nm^. This suggests that EMT can be dynamically regulated during the continued drug adaptation process and that there may be some level of plasticity even in the established resistant cell lines. Such plasticity may in addition to different laboratory routines and cell line evolution contribute to differing phenotypes among identical cell lines, as previously shown in HeLa and other cancer cell lines [55, 56].

Previous studies had further reported that the HCC4006^r^ERLO^1μm^ precursor cell line HCC4006^r^ERLO^0.5μm^ was characterised by Shc‐initiated RAS/RAF/MEK/ERK signalling and CIP2A‐mediated AKT activation and that MEK and AKT inhibitors inhibited the growth of this cell line in a similar way as the parental HCC4006 cell line [57, 58]. In contrast, HCC4006^r^ERLO^1μm^ displayed increased resistance to the MEK inhibitor trametinib relative to HCC4006, suggesting that survival of this cell line is not driven by MEK signalling. Moreover, while HCC4006^r^ERLO^1μm^ displayed similar sensitivity to the PI3K inhibitor LY294002 as HCC4006, it was cross‐resistant to the PI3K inhibitor alpelisib relative to HCC4006. Again, these discrepancies between HCC4006^r^ERLO^0.5μm^ and HCC4006^r^ERLO^1μm^ may indicate a pronounced level of cancer cell plasticity during the resistance formation process.

We further detected metabolic plasticity in HCC4006 and HCC827^r^GEFI^2μm^ during our project. Both cell lines temporarily displayed a Warburg metabolism, that is they did not consume oxygen despite its presence. The metabolic switch became apparent because confluent cell layers, which were confirmed to be viable by visual examination, stopped producing an MTT signal. The MTT assay measures oxidative phosphorylation in the mitochondria [20, 49, 50]. Hence, this indicated that the cells had shifted towards a Warburg metabolism and produced their ATP via glycolysis and subsequent lactic acid fermentation instead of via oxidative phosphorylation [47]. These findings illustrate the limitations of the MTT assay as viability assay in the context of metabolic changes [59] and emphasise the need for carefully selecting appropriate viability assays and the careful monitoring of the results that they produce for their accuracy. Notably, we have previously shown that drug–response data for individual drug/cell line combinations from the NCI60 screen was characterised by a very high level of variability [60]. Cancer cell line plasticity resulting in phenotypic changes during cultivation may contribute to these variations.

A lack of reproducibility is widely discussed in many scientific disciplines, including the cancer field [61, 62, 63, 64]. However, it is difficult to translate the awareness of cancer cell line plasticity, as reported here, into meaningful guidance for the improvement of data robustness. The NCI60 screen applies strictest quality measures, including following a standard operating procedure and using cell lines within a defined range of passages [65, 66, 67]. Nevertheless, its results are characterised by a remarkable level of variation [59, 68, 69]. Moreover, we here observed a reversible change in the cancer cell metabolism that occurred within a limited number of passages and was not reproducible. Hence, future research into cancer cell (line) plasticity will be needed to establish a better understanding of the underlying processes that can be used to inform improved experimental protocols.

We are not aware of studies that investigated cancer cell plasticity in the context of acquired resistance and acquired resistance formation in a similar way as we did here in cell lines in *in vivo* models. However, it is well established that numerous additional factors contribute to cancer cell plasticity in the *in vivo* situation, in particular different tumour microenvironments [68, 69, 70, 71, 72, 73]. Thus, it appears reasonable to assume that the cancer cell plasticity will, if anything, be more pronounced in an *in vivo* situation.

Cancer cell plasticity is, at least in part, regulated by epigenetic processes, including the activities of histone deacetylases (HDACs) [74]. Moreover, HDACs are involved in the regulation of signalling pathways, such as the PI3K/AKT and MEK/ERK signalling pathways, cancer cell metabolism and epithelial–mesenchymal transition (EMT), and all of these processes are relevant and interdependent in the context of drug resistance in cancer [59, 71, 74, 75, 76, 77, 78, 79]. However, the investigation of our panel of HDAC inhibitors did not suggest particular roles for certain HDACs in the project cell lines. Further prospective, longitudinal studies of cancer cell plasticity during and after cancer cell resistance acquisition will be required to establish a better understanding of the underlying processes, including their epigenetic regulation.

Generally, the determination of response profiles to cytotoxic anticancer drugs, kinase inhibitors and HDAC inhibitors resulted in complex patterns that were specific for each individual subline without obvious overlaps. This suggests that each resistance formation process follows its own unpredictable route. Notably, these findings are not only in line with other studies investigating drug‐resistant cancer cell lines, including those in which the same cell line was repeatedly adapted to the same drug in multiple experiments [17, 18, 36, 80, 81, 82, 83], but also with the complex evolutionary processes in cancer cells from lung cancer patients [84, 85, 86, 87, 88].

As a side aspect, we observed that drug response patterns differed when we did not directly correlate the drug effects but considered drug efficacy in the context of the respective clinically achievable (therapeutic) drug concentrations. Hence, direct drug response correlations may be of mechanistic relevance, but therapeutic concentrations need to be considered for translational approaches.

Notably, all resistant sublines remained sensitive or even displayed collateral sensitivity to at least one of the investigated drugs. This suggests that there are, in principle, effective treatments available for cancer cells that have acquired resistance to a certain drug. Currently, it is, however, usually not possible in a clinical setting to identify drugs that are effective against resistant cancer cells in a timely enough manner to benefit the patient. Therefore, further research will have to develop a mechanistic understanding of the vulnerabilities in resistant cancer cells that results in biomarkers that guide potential next‐line therapies to patients, who are likely to benefit from them and have run out of established therapy options.

In conclusion, we here introduce a novel panel of NSCLC cell lines with acquired resistance to EGFR kinase inhibitors. Drug response profiles differed significantly between the resistant sublines, suggesting that each resistance formation process follows an individual, unpredictable path. The comparison of some of the sublines with precursor cell lines that had been previously characterised at a lower resistance level indicated a substantial level of phenotypic heterogeneity during the ongoing resistance formation process. Moreover, HCC4006 and HCC827^r^GEFI^2μm^ displayed metabolic plasticity during the course of our experiments. This suggests that cancer cells are subject to a continuous plasticity that affects their drug sensitivity profiles and may contribute to the variability in cell line phenotypes observed between different laboratories and also in intra‐laboratory experiments [55, 56, 60]. Future research will have to establish a detailed understanding of these dynamic processes that can be translated into biomarker‐guided strategies that guide effective therapies to patients with therapy‐refractory disease for whom currently no standard treatment options are available.

## Conflict of interest

The authors declare no conflict of interest.

## Peer review

The peer review history for this article is available at https://www.webofscience.com/api/gateway/wos/peer‐review/10.1002/2211‐5463.70076.

## Author contributions

TVN, MNW and MM conceived and designed the study. TVN and JC acquired data. TVN, CWG, JC, MNW and MM analysed and curated data. IO and MDG provided materials. TVN and MM drafted the work. All authors revised the work and approved the submitted version.

## Supporting information


**Fig. S1.** Dose–response curves of HCC827 and HCC4006 and their EGFR tyrosine kinase inhibitor‐resistant sublines to different EGFR tyrosine kinase inhibitors.
**Fig. S2.** Dose–response curves of HCC827 and HCC4006 and their EGFR tyrosine kinase inhibitor‐resistant sublines to different cytotoxic anticancer drugs.
**Fig. S3.** Dose–response curves of HCC827 and HCC4006 and their EGFR tyrosine kinase inhibitor‐resistant sublines to different kinase inhibitors.
**Fig. S4.** Effects of kinase inhibitors on the sensitivity of erlotinib‐adapted sublines to erlotinib.
**Fig. S5.** Effects of kinase inhibitors on the sensitivity of gefitinib‐adapted sublines to gefitinib.
**Fig. S6.** Effects of kinase inhibitors on the sensitivity of afatinib‐adapted sublines to afatinib.
**Fig. S7.** Determination of IC50 and IC90 value of vincristine in HCC4006 and HCC827 and their EGFR tyrosine kinase‐adapted sublines in the presence or absence of the ABCB1 inhibitor zosuquidar.
**Fig. S8.** Summary of high‐resolution respirometry results of HCC827, HCC4006 and respective EGFR tyrosine kinase inhibitor‐adapted sublines.
**Table S1.** Doubling times of HCC827 and HCC4006 and their EGFR tyrosine kinase inhibitor‐adapted sublines in the absence and presence of drug.
**Table S2.** Sensitivity of HCC827, HCC4006 and their EGFR tyrosine kinase inhibitor‐resistant sublines to EGFR tyrosine kinase inhibitors.
**Table S3.** Sensitivity of HCC827, HCC4006 and their EGFR tyrosine kinase inhibitor‐resistant sublines to cytotoxic anticancer drugs.
**Table S4.** Sensitivity of HCC827, HCC4006 and their EGFR tyrosine kinase inhibitor‐resistant sublines to different kinase inhibitors.
**Table S5.** Determination of the IC25 and IC50 values for different kinase inhibitors in HCC827, HCC4006 and their EGFR tyrosine kinase inhibitor‐resistant sublines to different kinase inhibitors.
**Table S6.** Impact of different kinase inhibitors on the sensitivity of EGFR tyrosine kinase‐adapted HCC827 and HCC4006 sublines to their respective drugs of adaptation.
**Table S7.** HDAC inhibitor concentrations inhibit HDACs with some level of specificity.
**Table S8.** Effect of HDAC inhibitors on the viability of the project cell lines.

## Data Availability

All data are provided in the manuscript and the supplements.
