# Supplementary material for: Phenotypic plasticity in a novel set of EGFR tyrosine kinase inhibitor‐adapted non‐small cell lung cancer cell lines
Source: FEBS Open Bio. 2025 Jun 26;15(11):1854–73. doi: 10.1002/2211-5463.70076 (PMC12582973; doi:10.1002/2211-5463.70076)
Supplement: Supplementary file 1 — Fig. S1. Dose–response curves of HCC827 and HCC4006 and their EGFR tyrosine kinase inhibitor‐resistant sublines to different EGFR tyrosine kinase inhibitors. Fig. S2. Dose–response curves of HCC827 and HCC4006 and their EGFR tyrosine kinase inhibitor‐resistant sublines to different cytotoxic anticancer drugs. Fig. S3. Dose–response curves of HCC827 and HCC4006 and their EGFR tyrosine kinase inhibitor‐resistant sublines to different kinase inhibitors. Fig. S4. Effects of kinase inhibitors on the sensitivity of erlotinib‐adapted sublines to erlotinib. Fig. S5. Effects of kinase inhibitors on the sensitivity of gefitinib‐adapted sublines to gefitinib. Fig. S6. Effects of kinase inhibitors on the sensitivity of afatinib‐adapted sublines to afatinib. Fig. S7. Determination of IC50 and IC90 value of vincristine in HCC4006 and HCC827 and their EGFR tyrosine kinase‐adapted sublines in the presence or absence of the ABCB1 inhibitor zosuquidar. Fig. S8. Summary of high‐resolution respirometry results of HCC827, HCC4006 and respective EGFR tyrosine kinase inhibitor‐adapted sublines. Table S1. Doubling times of HCC827 and HCC4006 and their EGFR tyrosine kinase inhibitor‐adapted sublines in the absence and presence of drug. Table S2. Sensitivity of HCC827, HCC4006 and their EGFR tyrosine kinase inhibitor‐resistant sublines to EGFR tyrosine kinase inhibitors. Table S3. Sensitivity of HCC827, HCC4006 and their EGFR tyrosine kinase inhibitor‐resistant sublines to cytotoxic anticancer drugs. Table S4. Sensitivity of HCC827, HCC4006 and their EGFR tyrosine kinase inhibitor‐resistant sublines to different kinase inhibitors. Table S5. Determination of the IC25 and IC50 values for different kinase inhibitors in HCC827, HCC4006 and their EGFR tyrosine kinase inhibitor‐resistant sublines to different kinase inhibitors. Table S6. Impact of different kinase inhibitors on the sensitivity of EGFR tyrosine kinase‐adapted HCC827 and HCC4006 sublines to their respective drugs of adaptation. Tabl [file FEB4-15-1854-s001.docx]

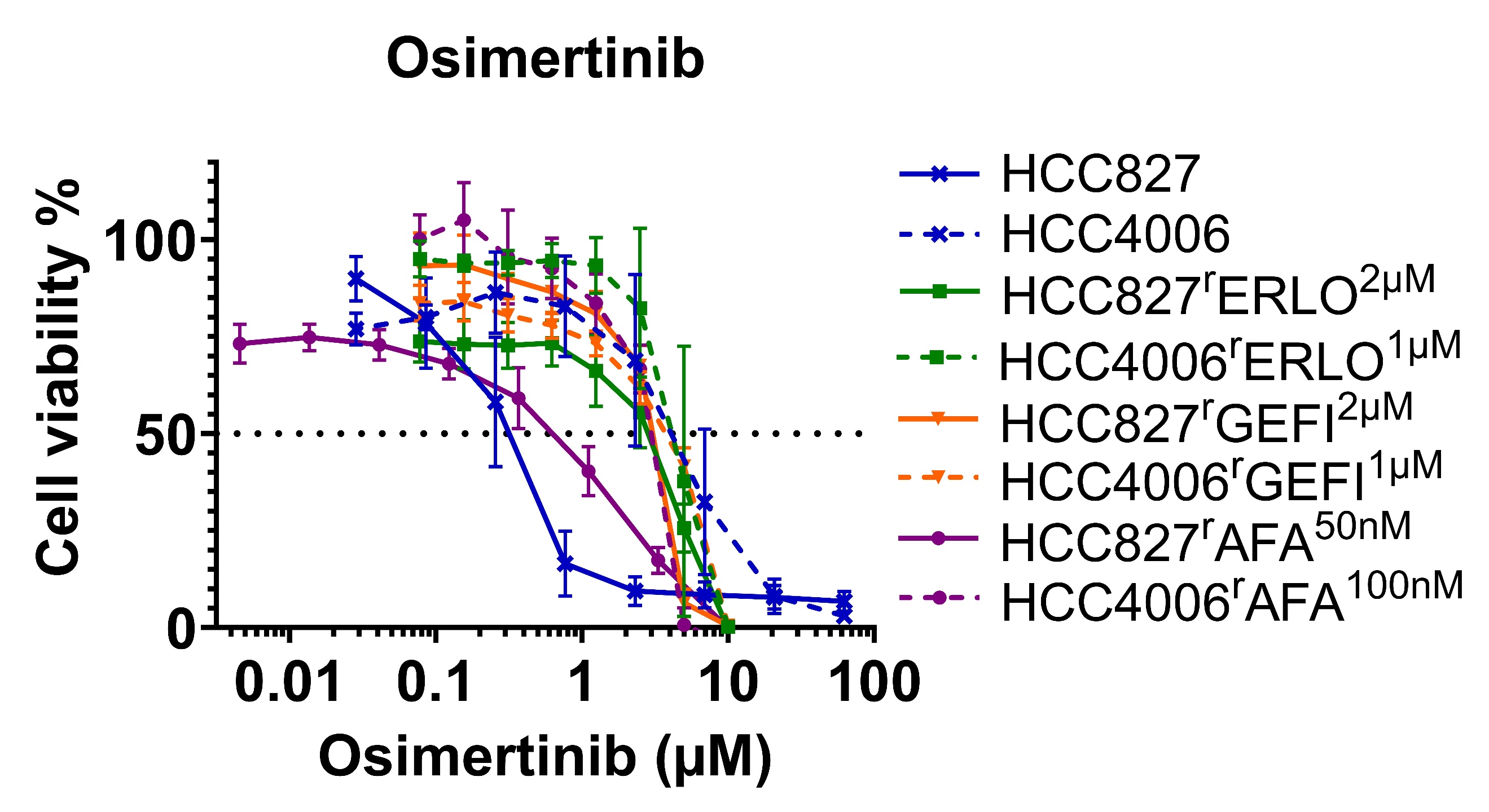

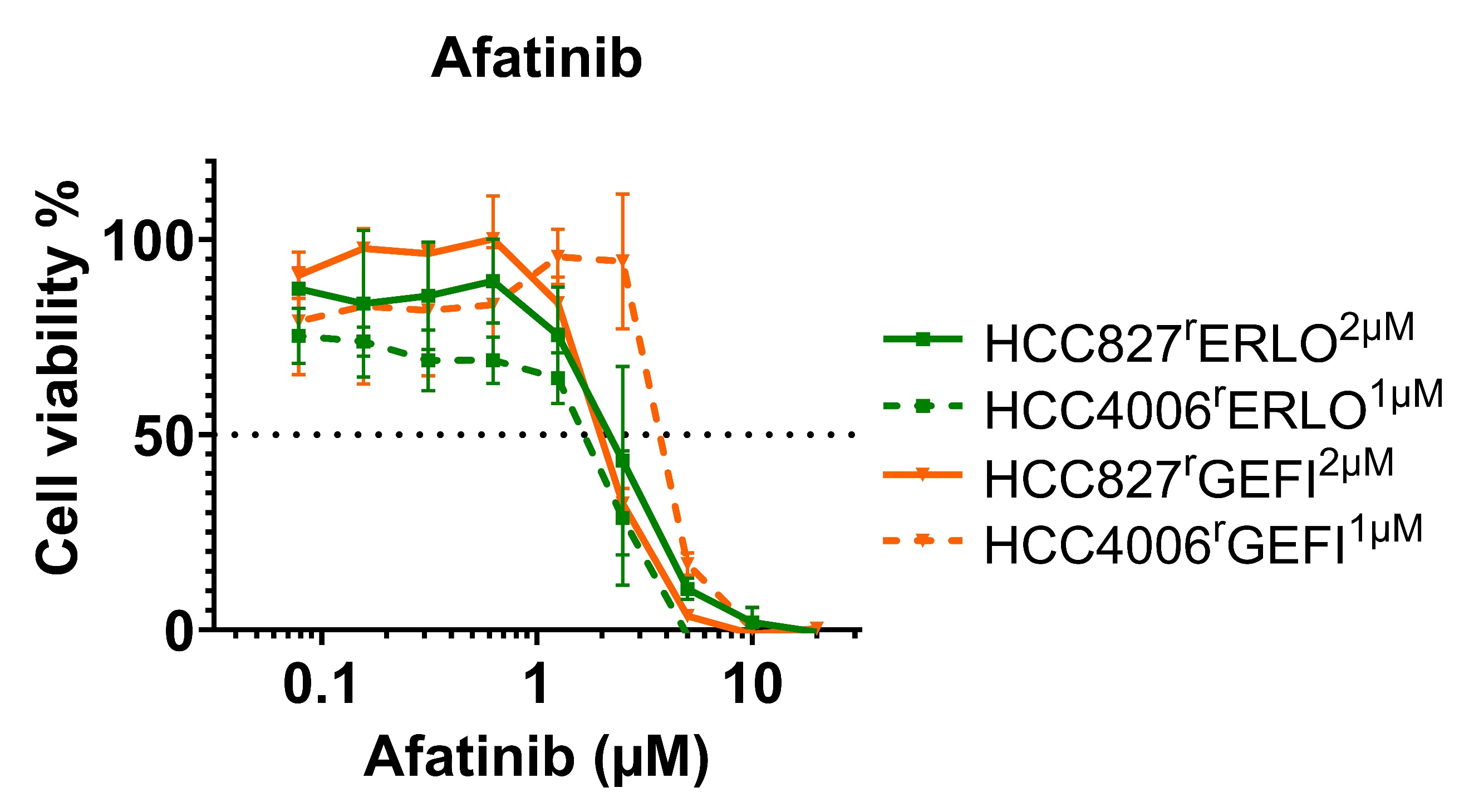

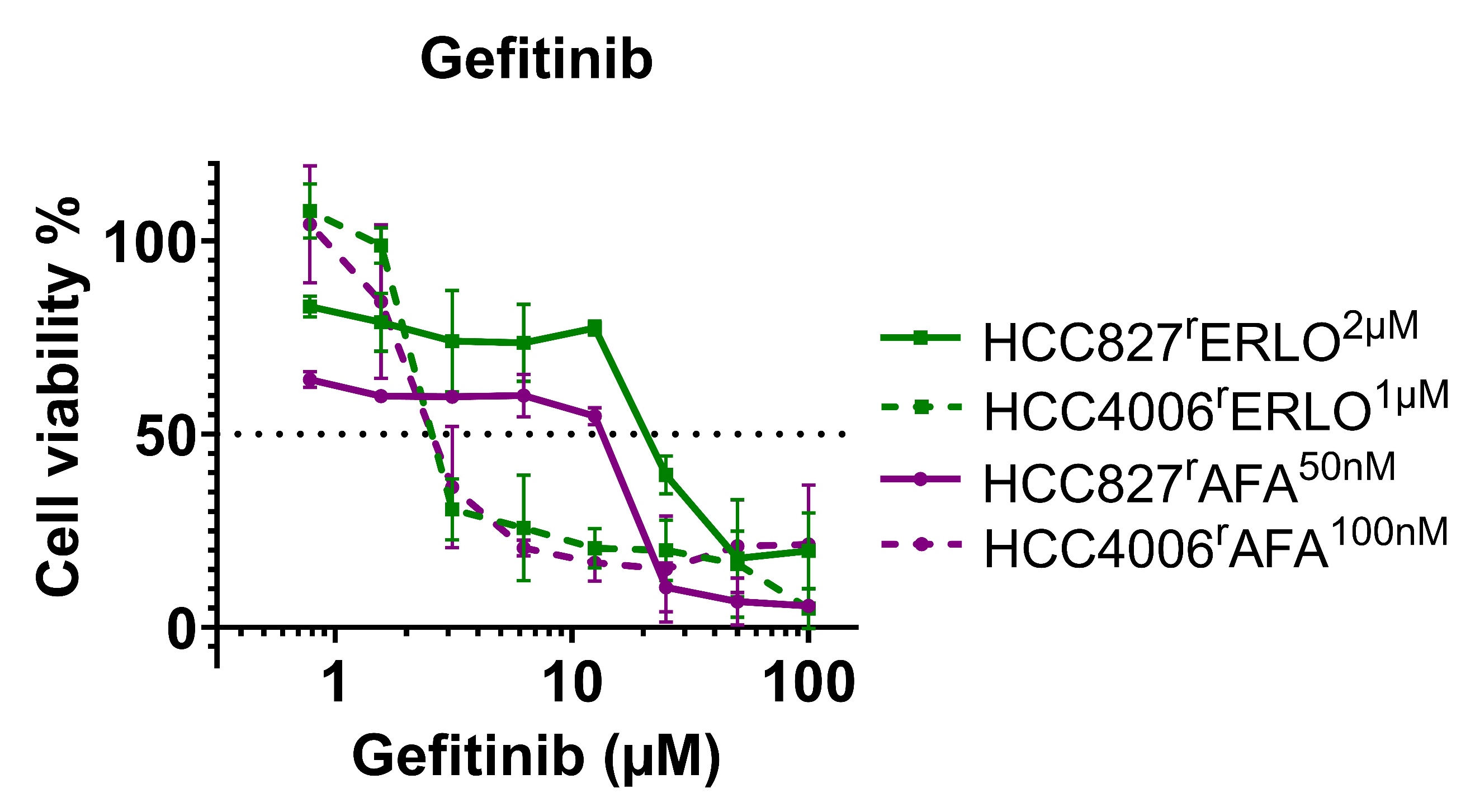

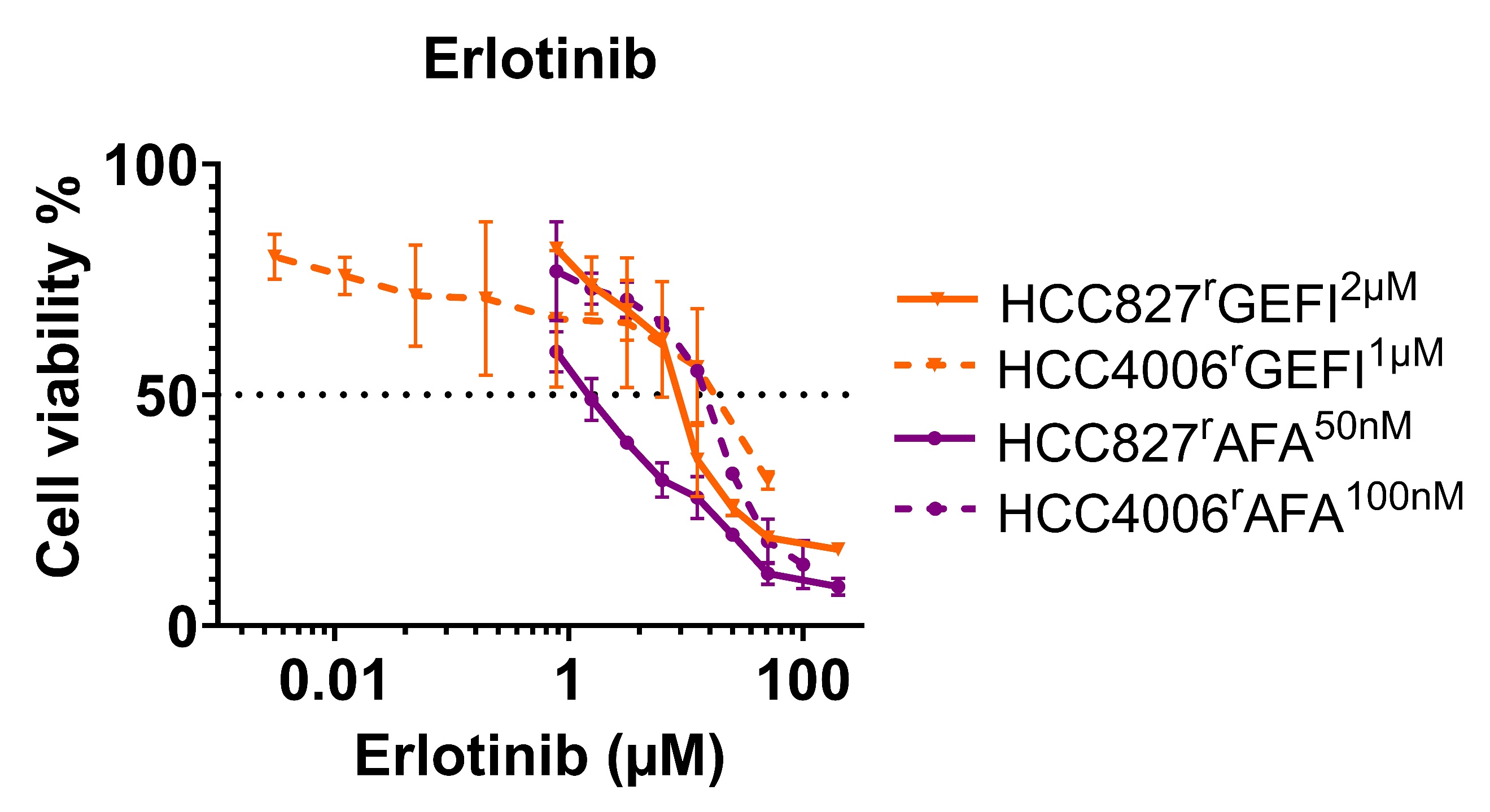


**Supplementary Figure 1. Dose response curves of HCC827 and HCC4006 and their EGFR tyrosine kinase inhibitor-resistant sublines to different EGFR tyrosine kinase inhibitors.** Data points represent mean of three independent biological repeats ± S.D, as determined by MTT assay after a 120h incubation period.


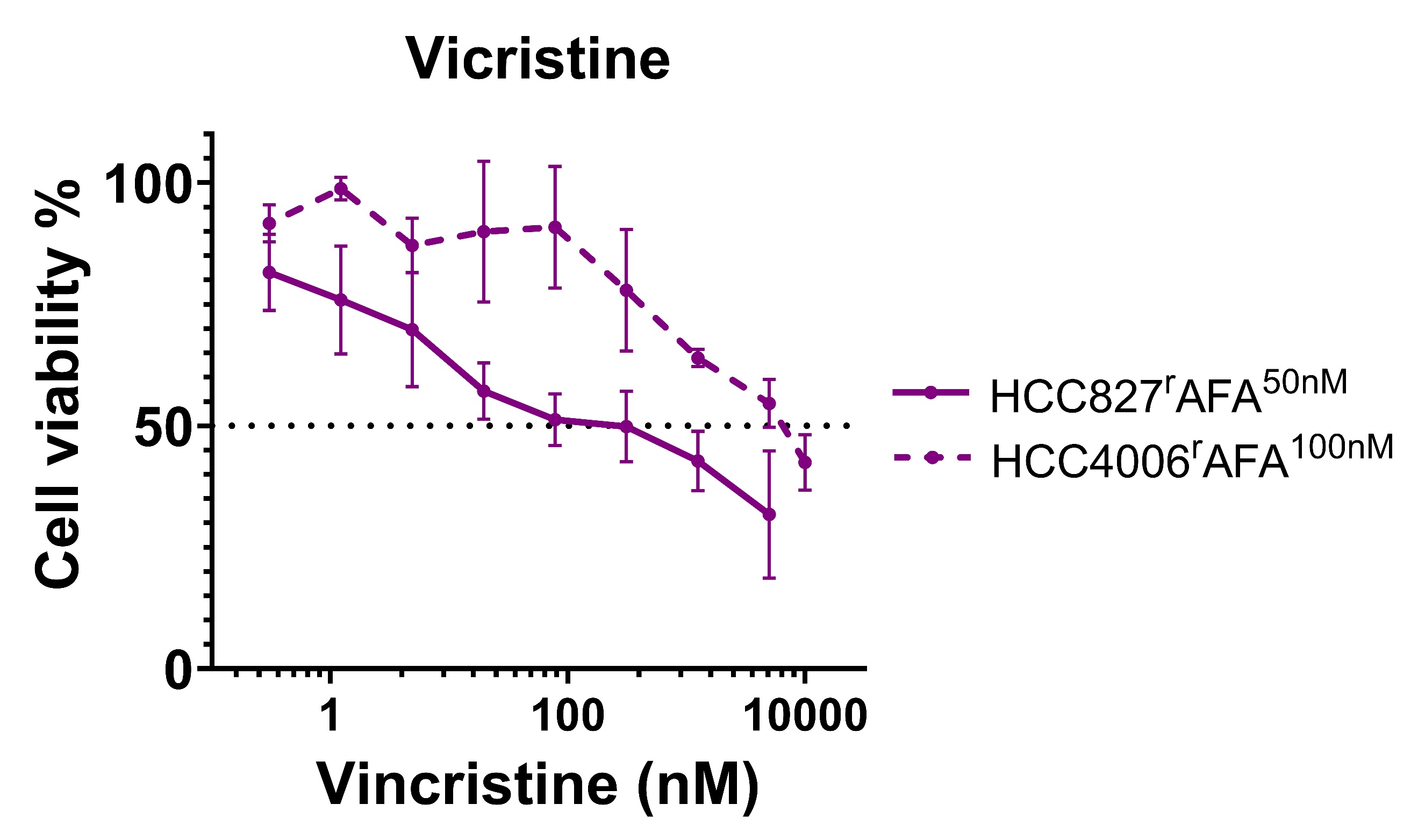

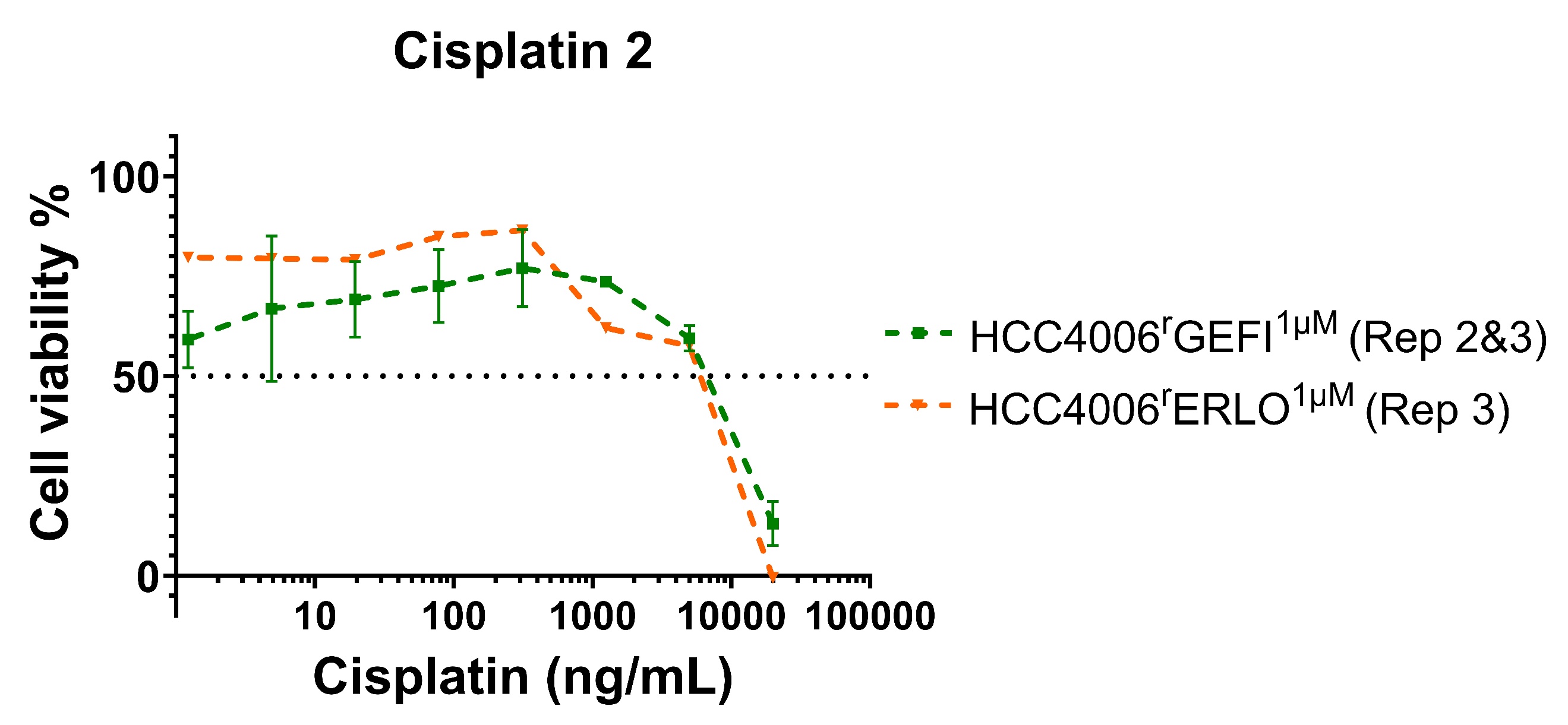

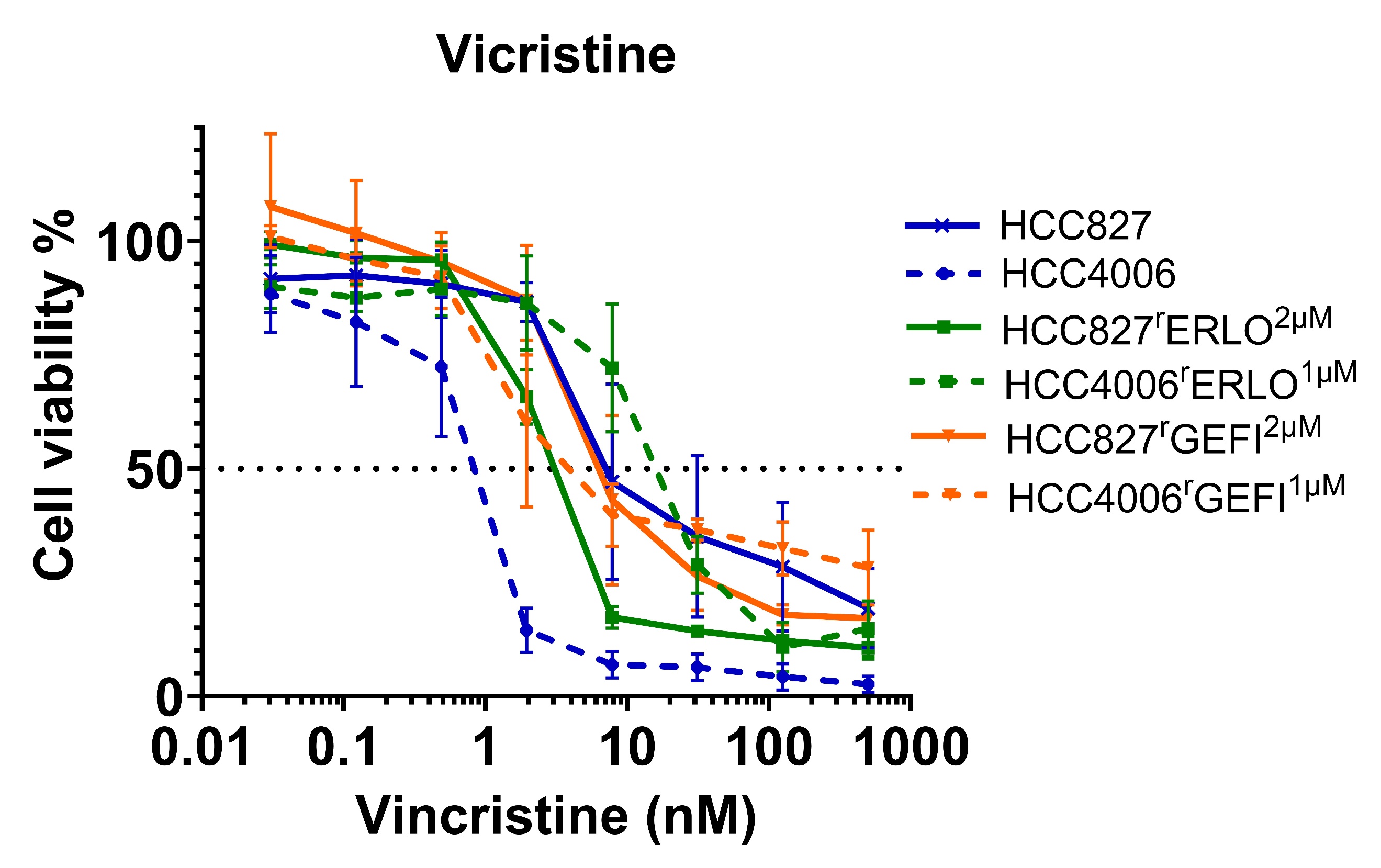

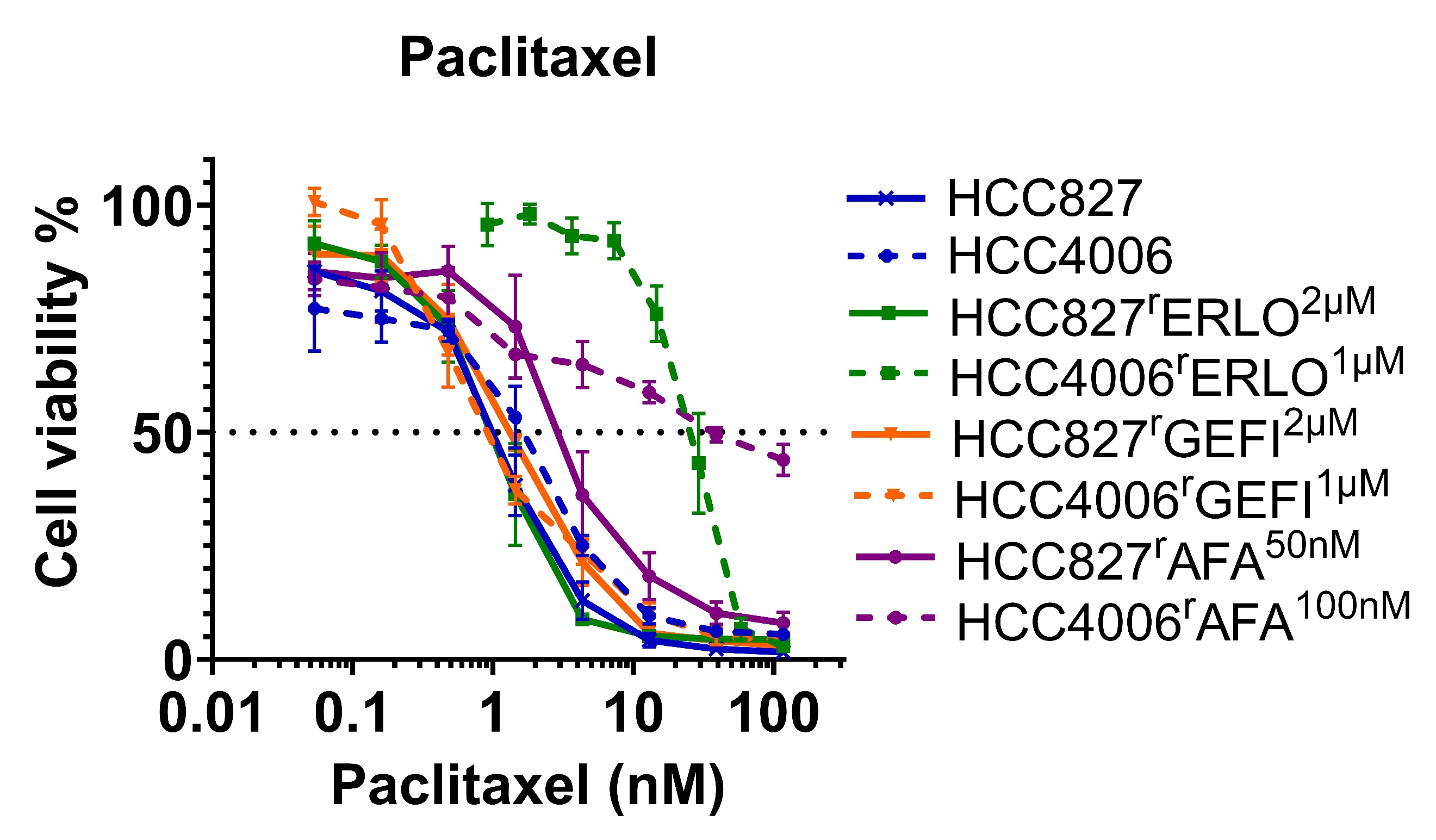

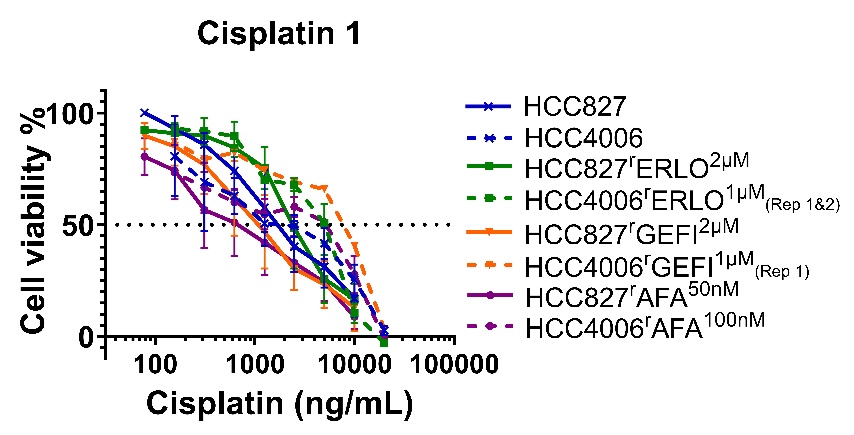
**Supplementary Figure 2. Dose response curves of HCC827 and HCC4006 and their EGFR tyrosine kinase inhibitor-resistant sublines to different cytotoxic anti-cancer drugs.** Data points represent mean of three independent biological repeats (for cisplatin, three biological repeats plotted in two different graphs) ± S.D, as determined by MTT assay after a 120h incubation period.


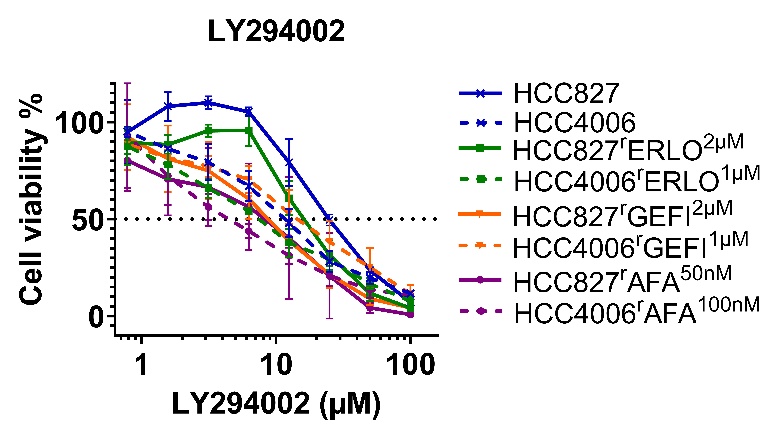

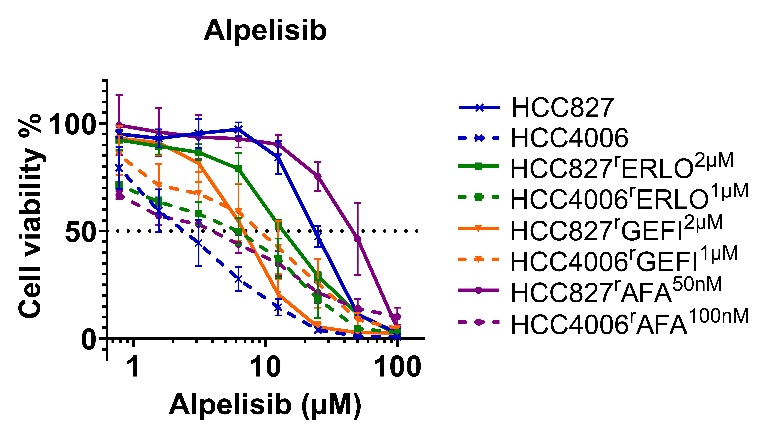

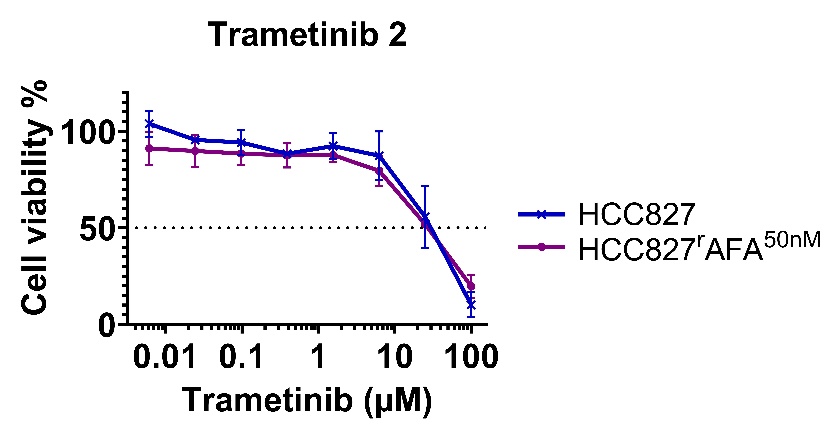

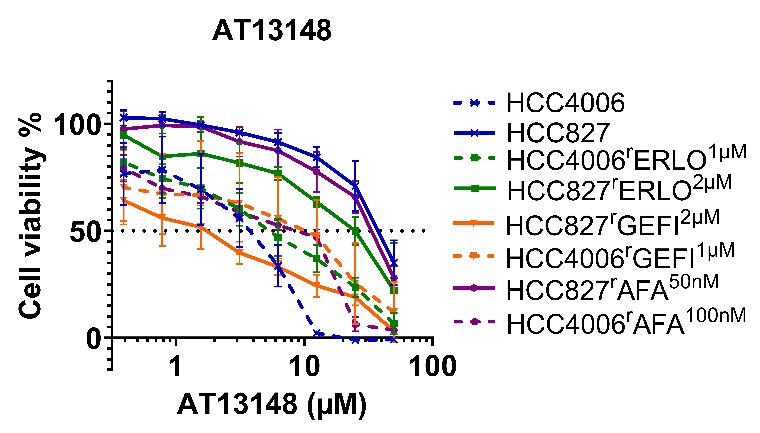

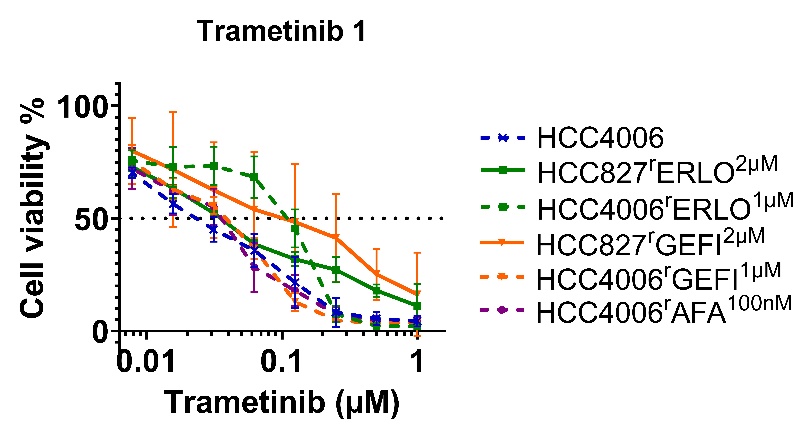

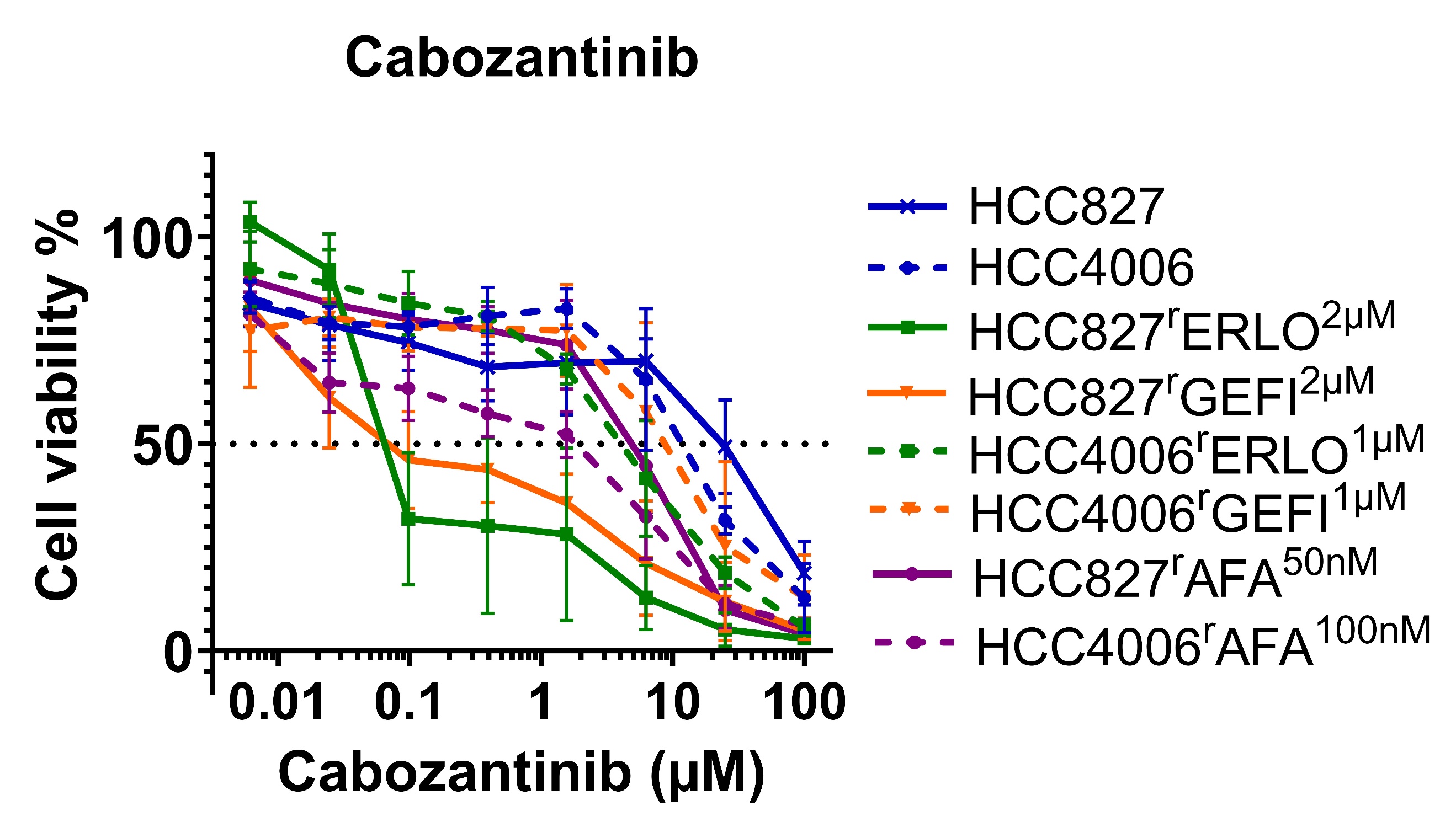


**Supplementary Figure 3. Dose response curves of HCC827 and HCC4006 and their EGFR tyrosine kinase inhibitor-resistant sublines to different kinase inhibitors.** Data points represent mean of three independent biological repeats (for trametinib three biological repeats plotted in two different graphs) ± S.D, as determined by MTT assay after a 120h incubation period.


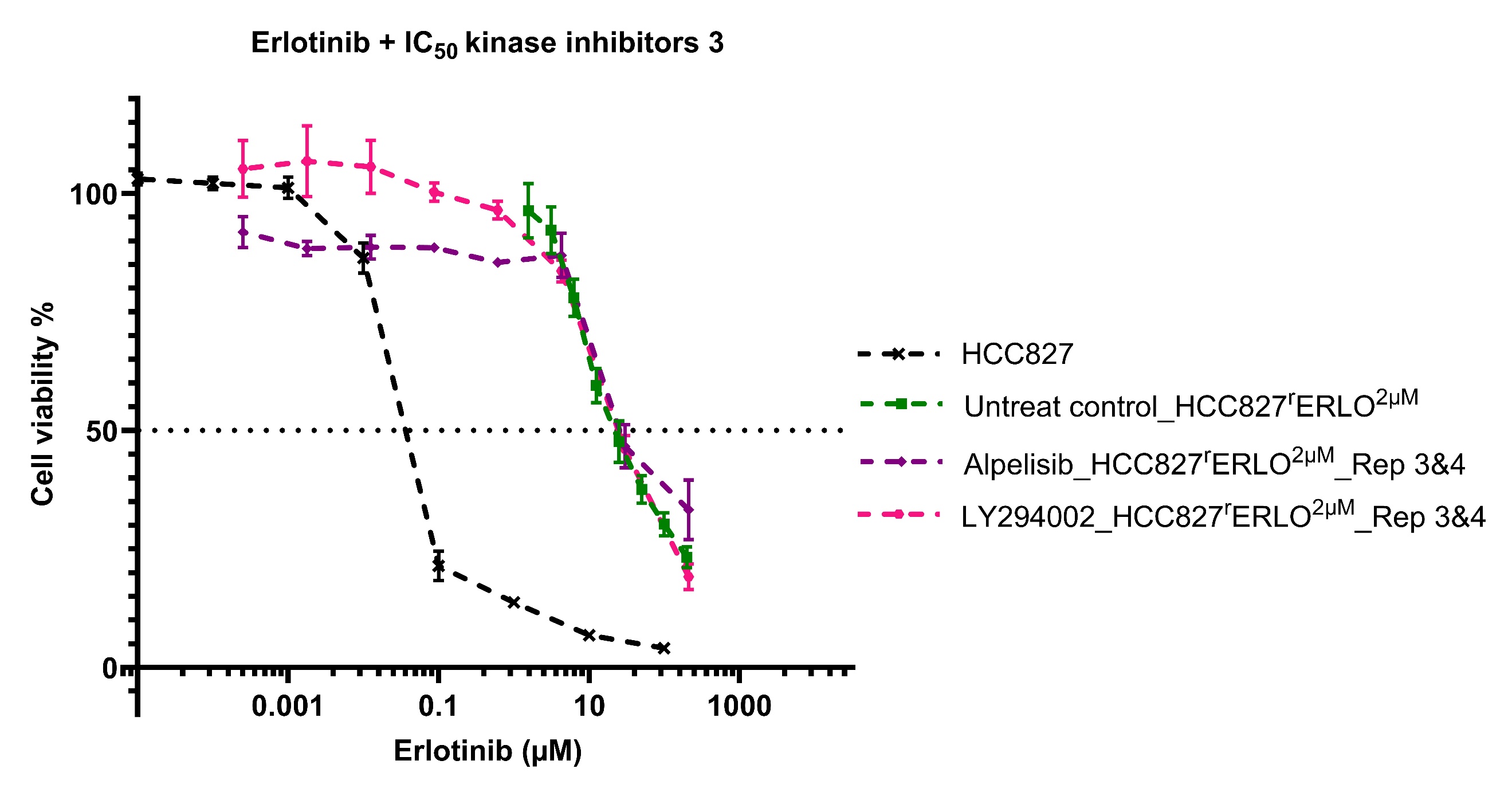

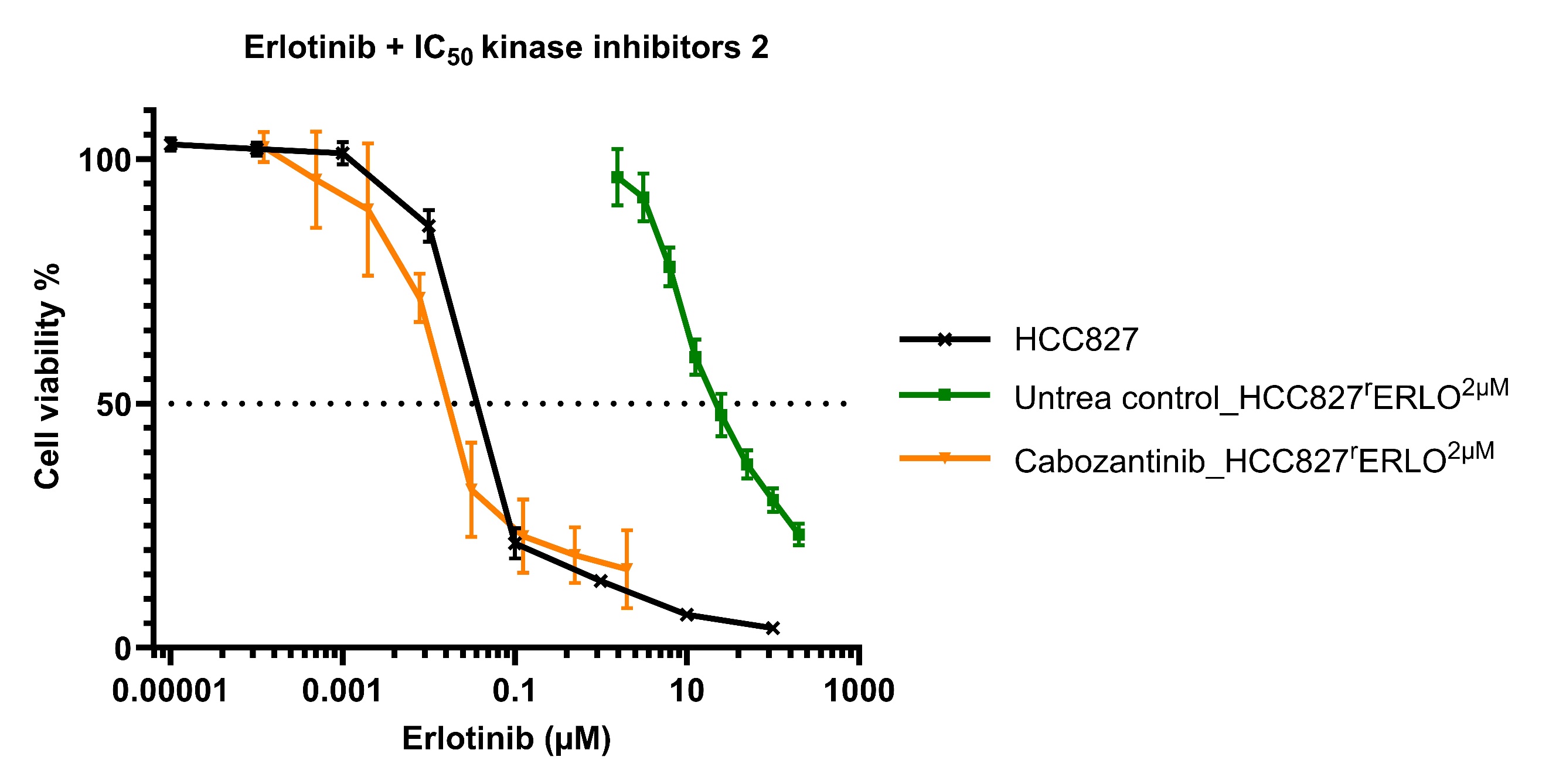

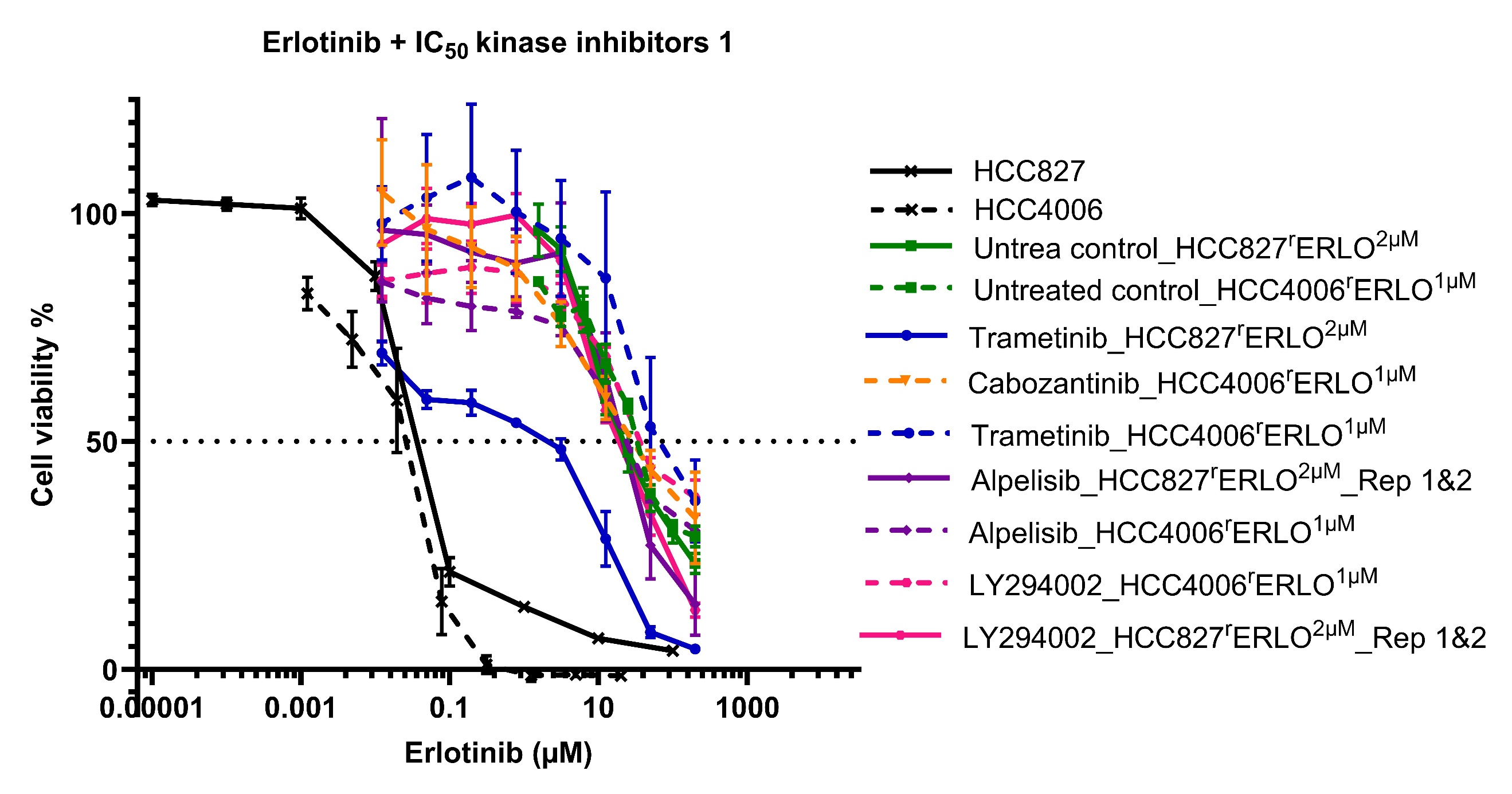


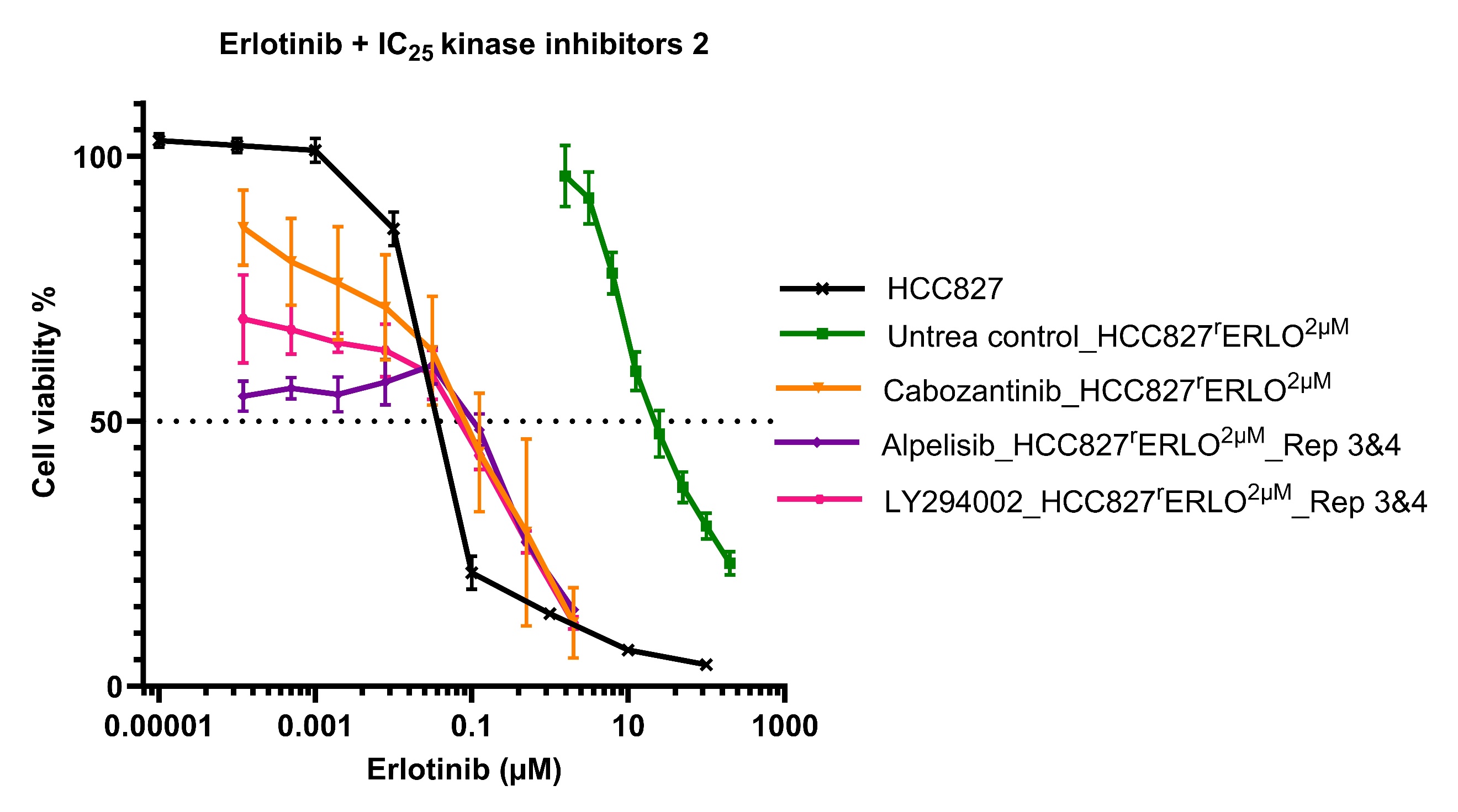

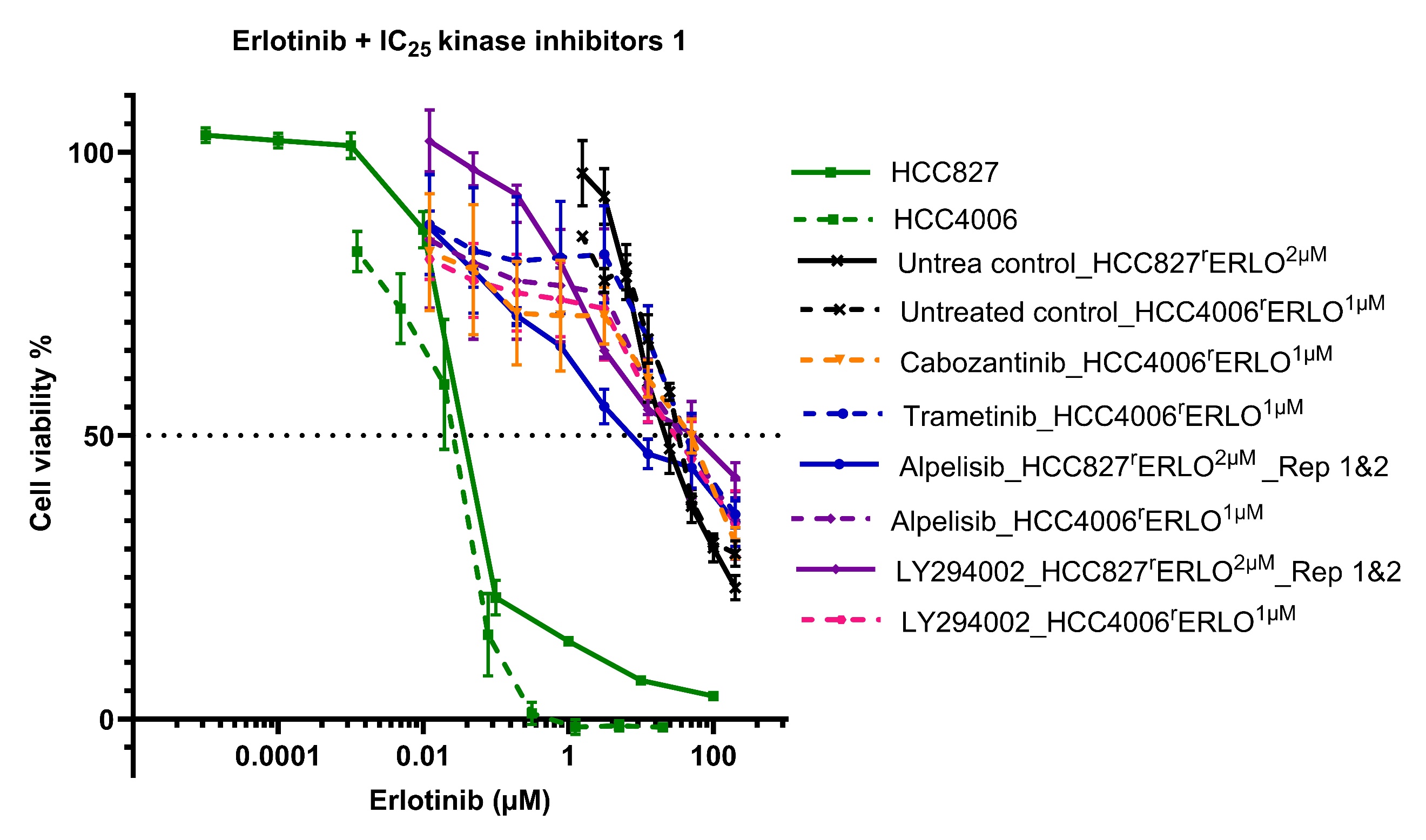
**Supplementary Figure 4. Effects of kinase inhibitors on the sensitivity of erlotinib-adapted sublines to erlotinib.** Data points represent mean of three independent biological repeats (n=4 for alpelisib and LY294002) ± S.D, as determined by MTT assay after a 120h incubation period.


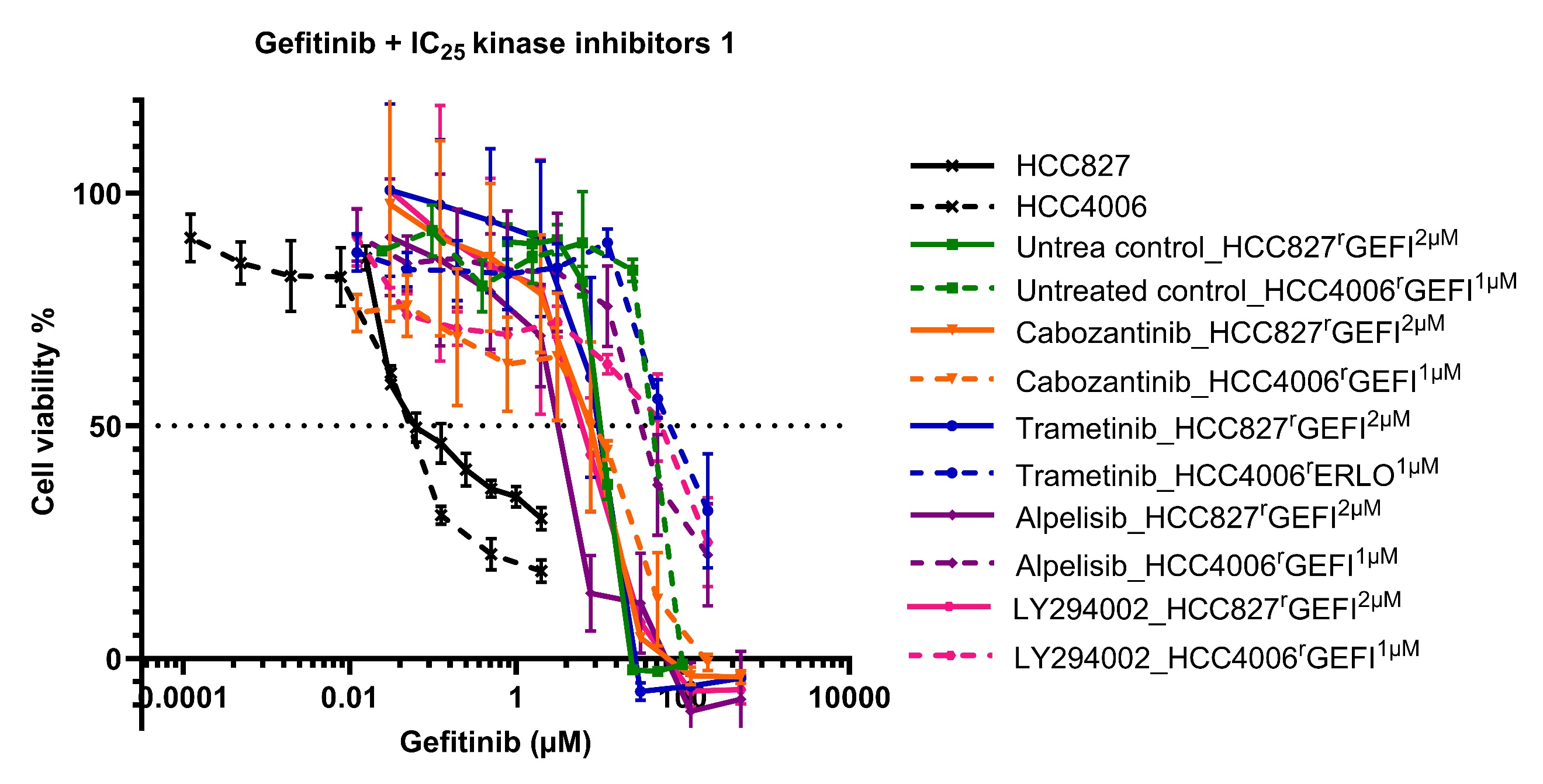

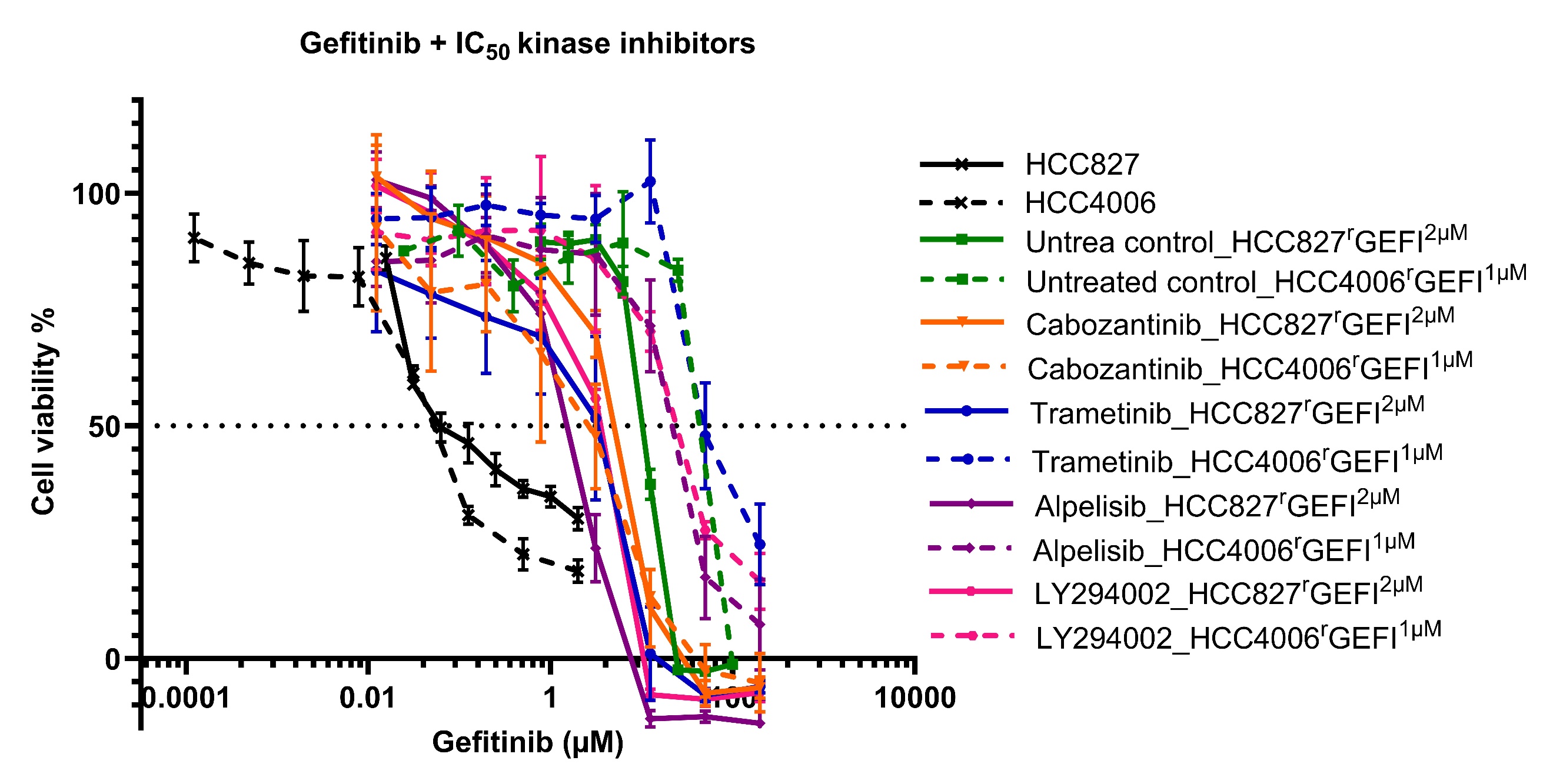


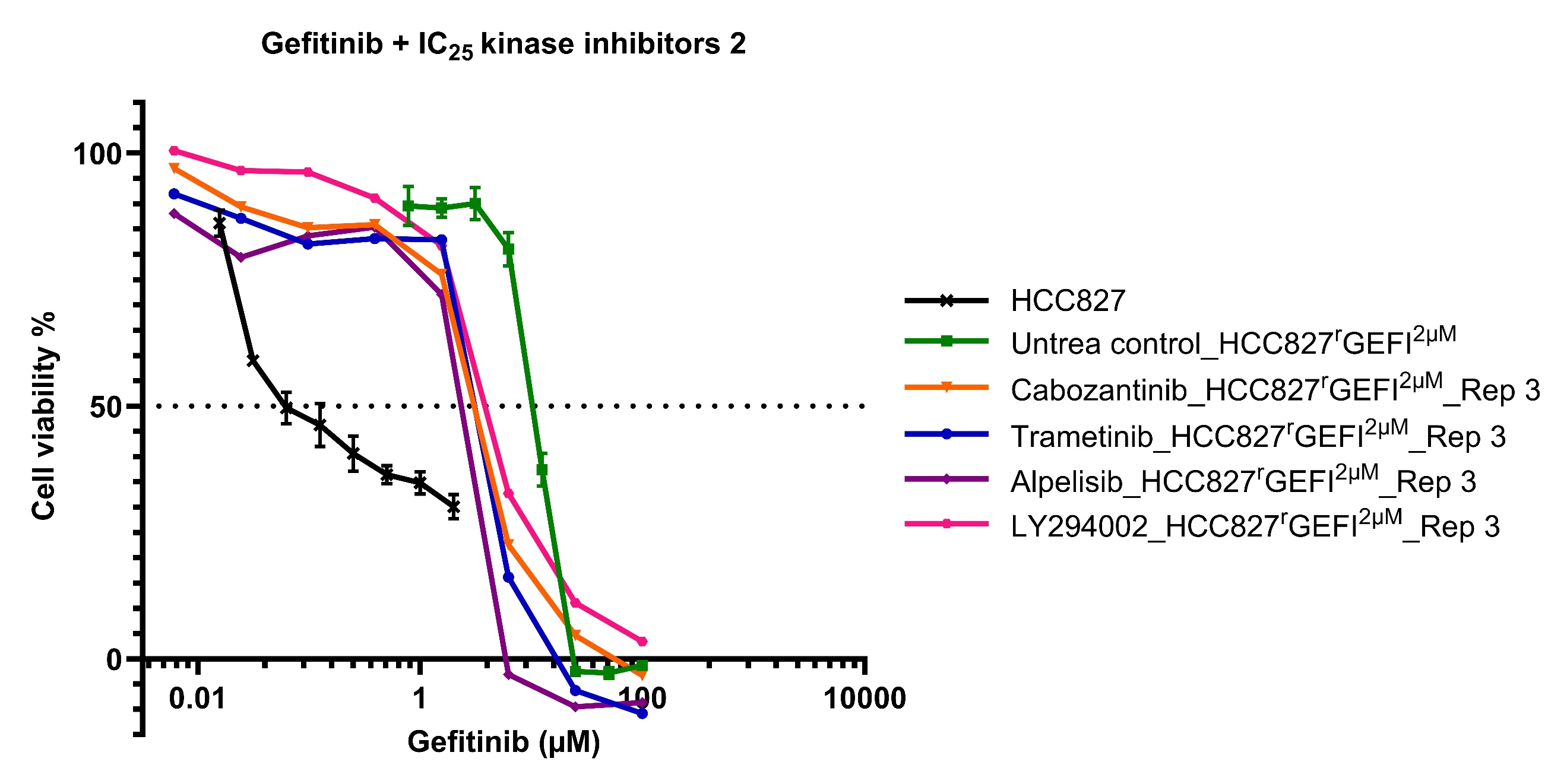
**Supplementary Figure 5. Effects of kinase inhibitors on the sensitivity of gefitinib-adapted sublines to gefitinib.** Data points represent mean of three independent biological repeats ± S.D, as determined by MTT assay after a 120h incubation period.


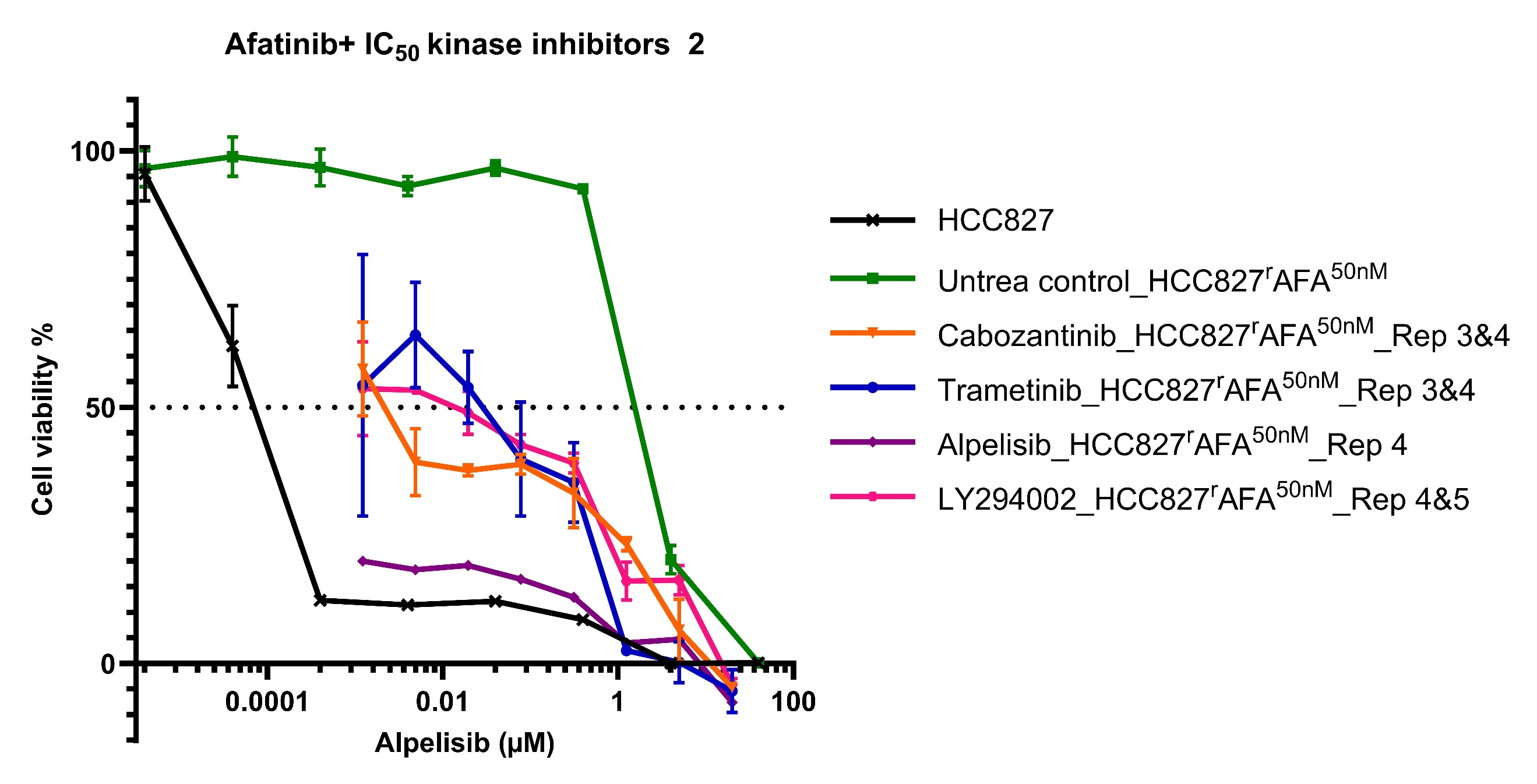

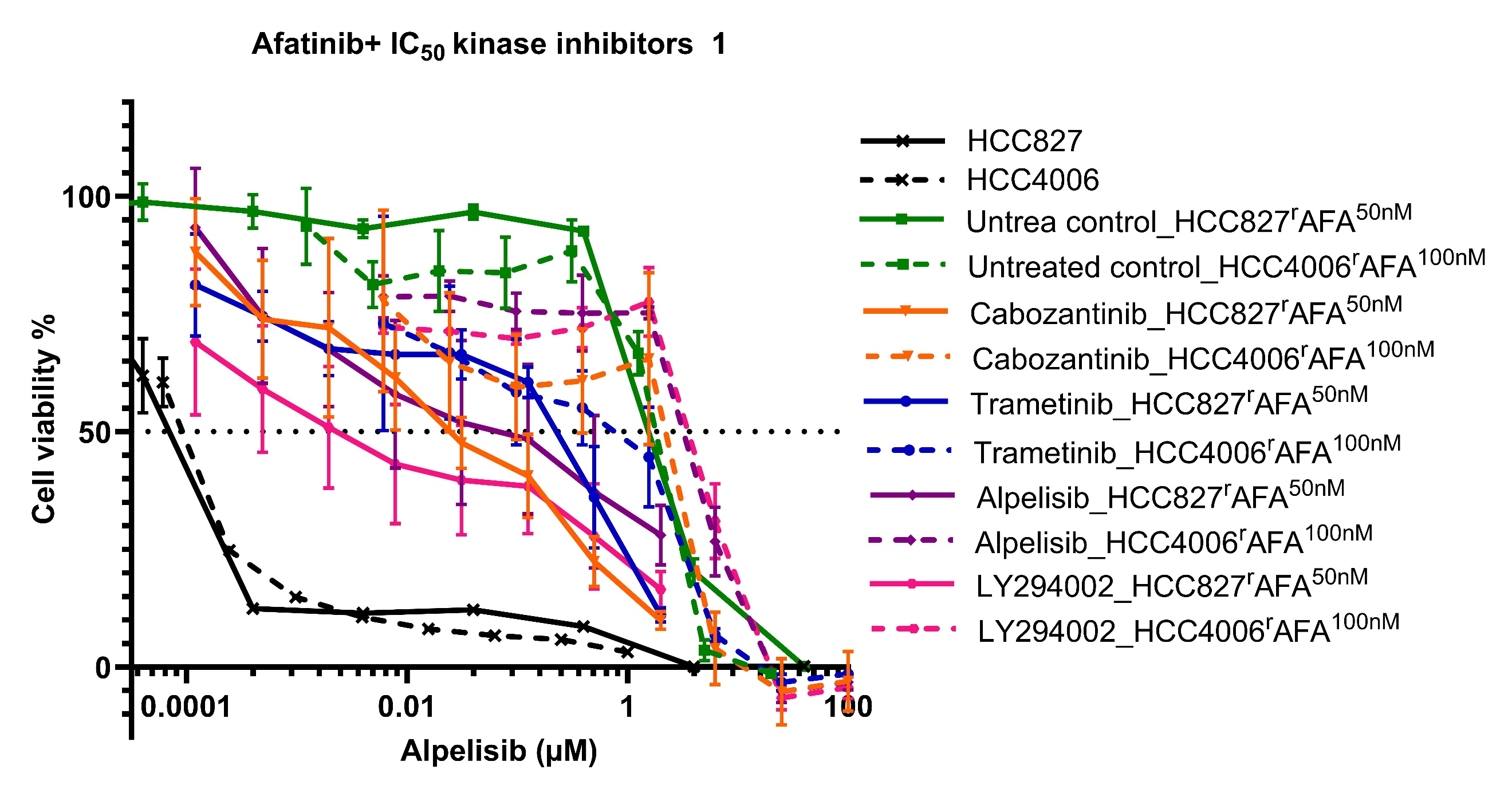


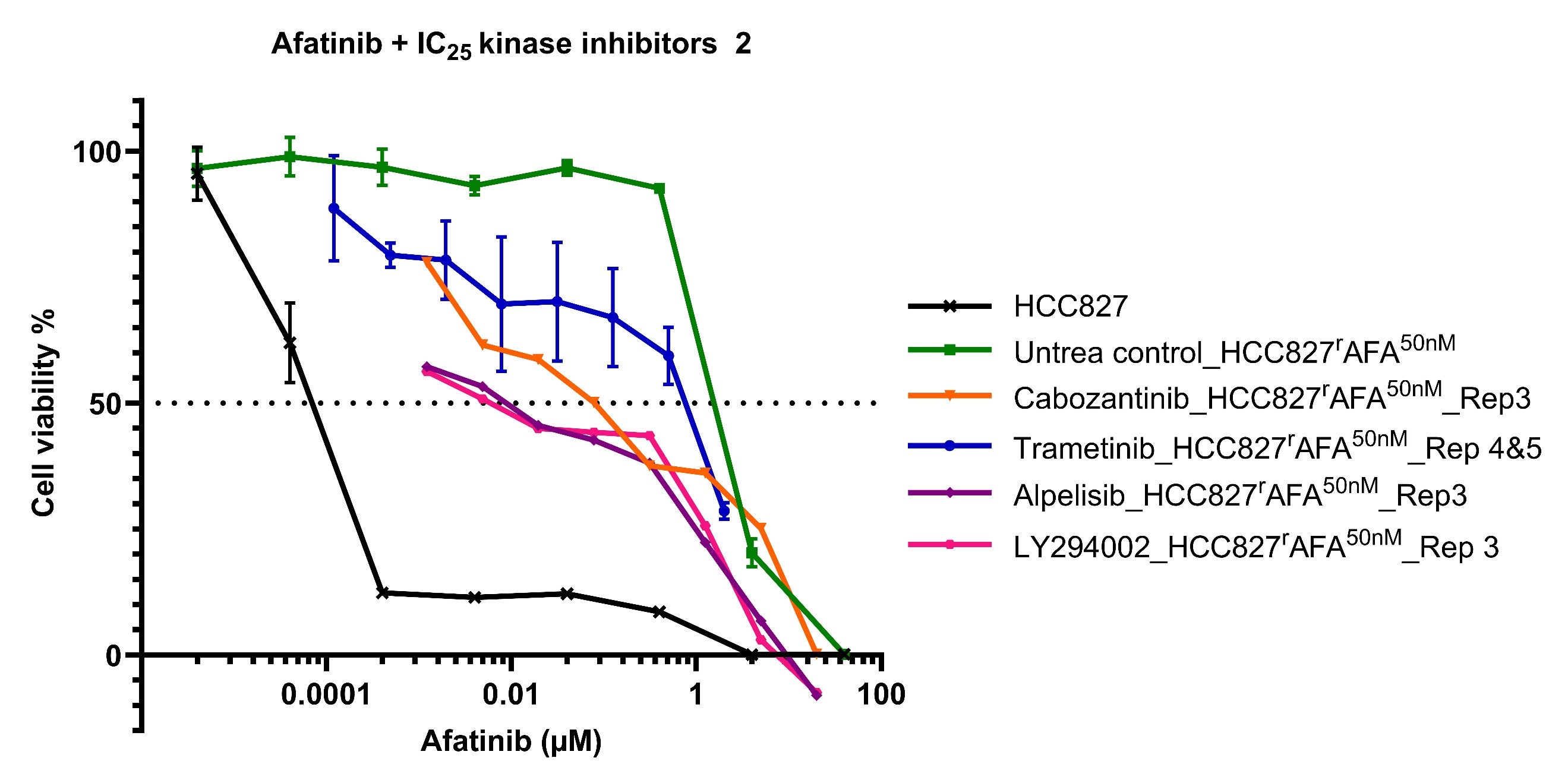

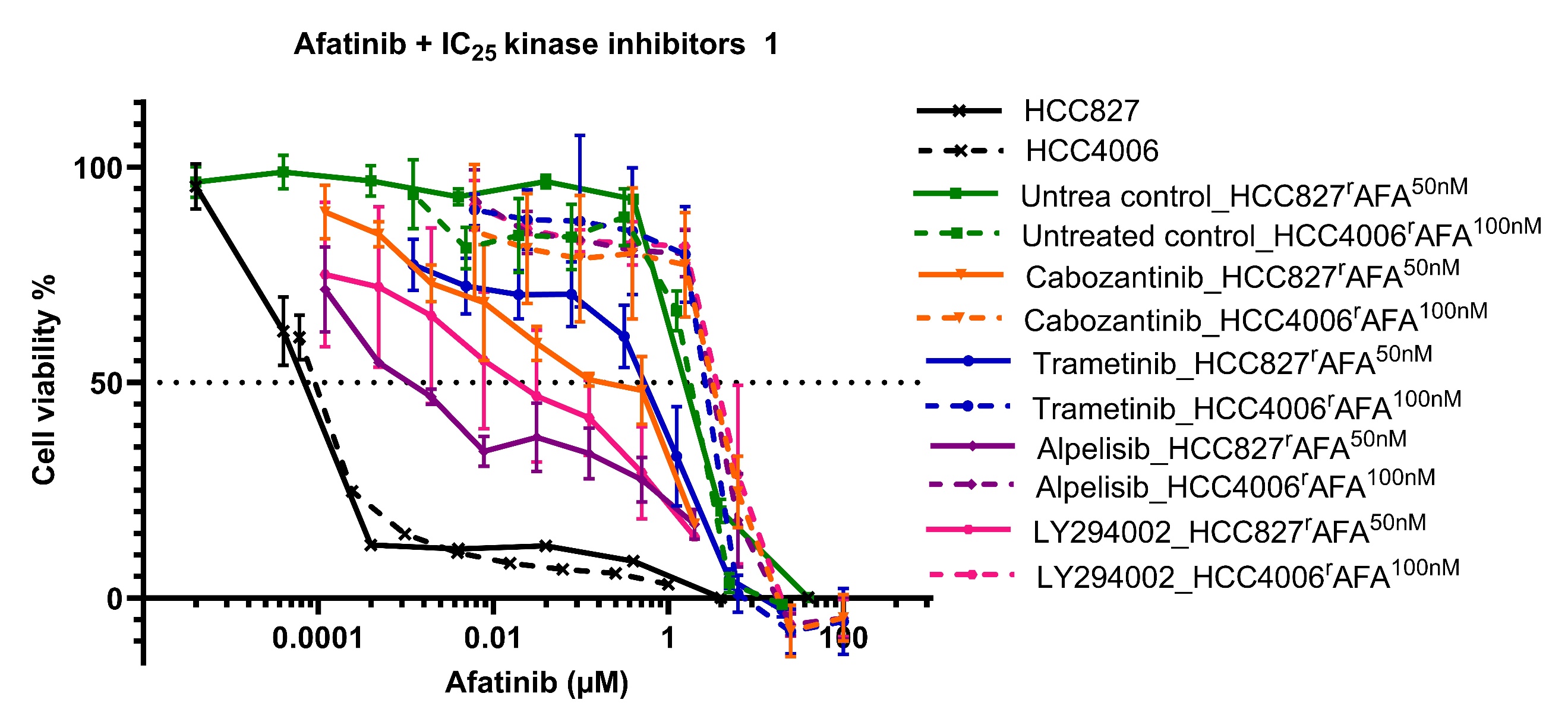


**Supplementary Figure 6. Effects of kinase inhibitors on the sensitivity of afatinib-adapted sublines to afatinib.** Data points represent mean of three independent biological repeats (n=5 for trametinib) ± S.D, as determined by MTT assay after a 120h incubation period.


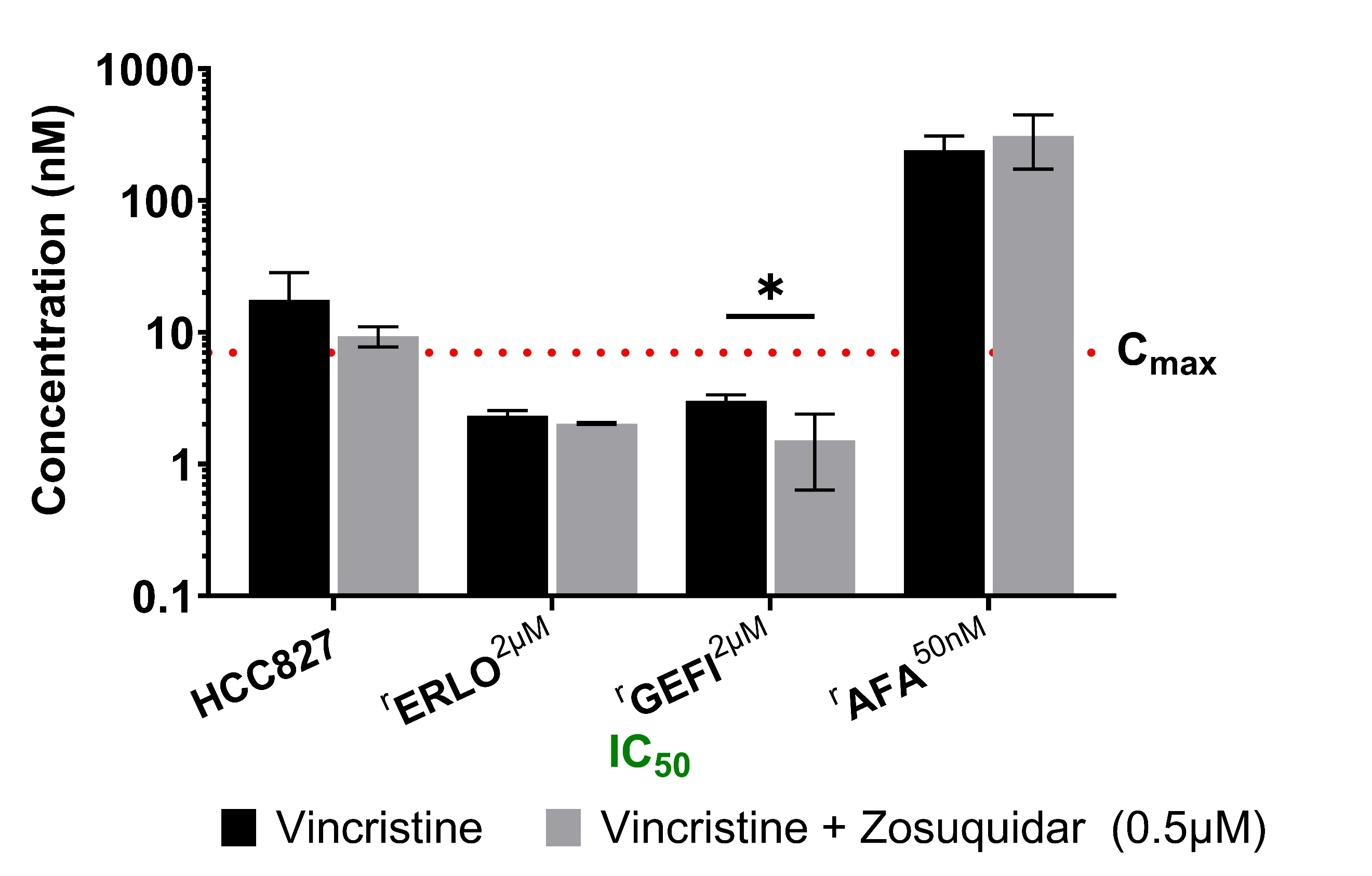
**A.**


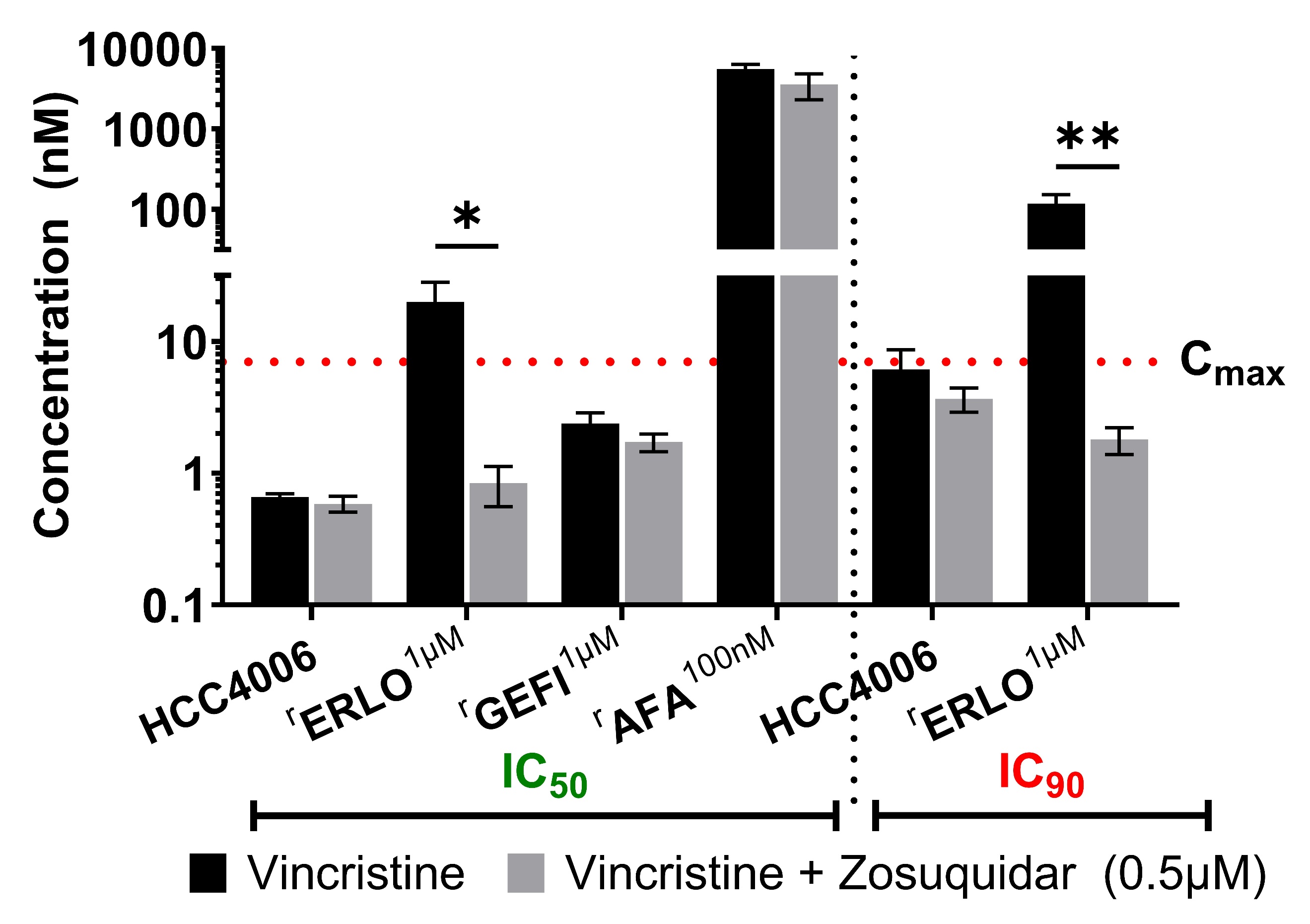
**B.**

**Supplementary Figure 7. Determination of IC_50_ and IC_90_ value of vincristine in HCC4006 and HCC827 and their EGFR tyrosine kinase-adapted sublines in the presence or absence of the ABCB1 inhibitor zosuquidar.** Drug response was determined by MTT assay after a 120h incubation period. IC_50_ values were calculated using Calcusyn (Version 1.1, Biosof 1996). The data are means of three independent biological repeats ± S.D. Differences were analysed for statistical significance (p<0.05) by student’s t-test. *p<0.05, **p<0.01**. C_max_ – maximum therapeutic plasma concentration, IC_50_ - half maximal inhibitory concentrations, IC_90_ - concentration that inhibit cell viability by 90%.


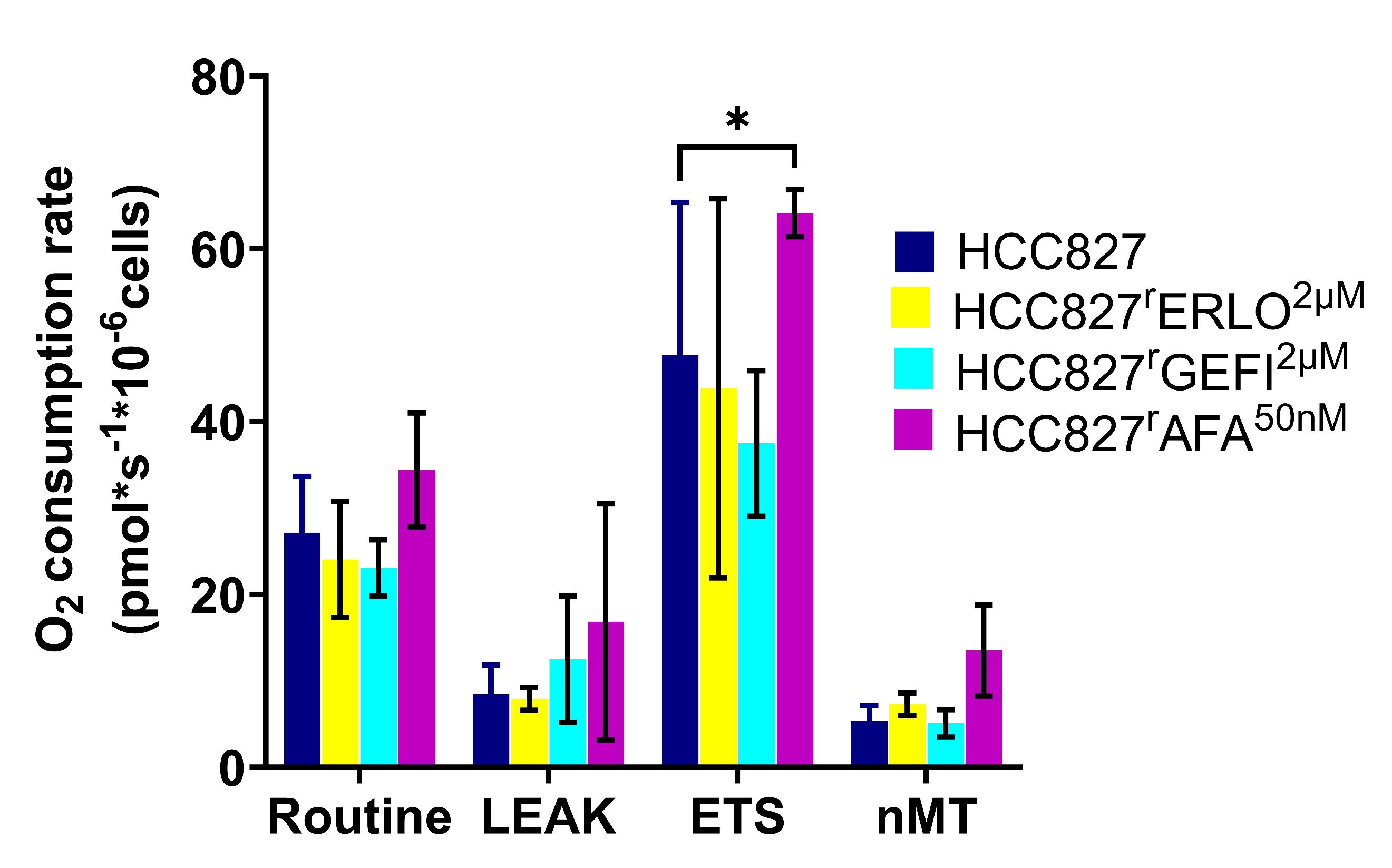

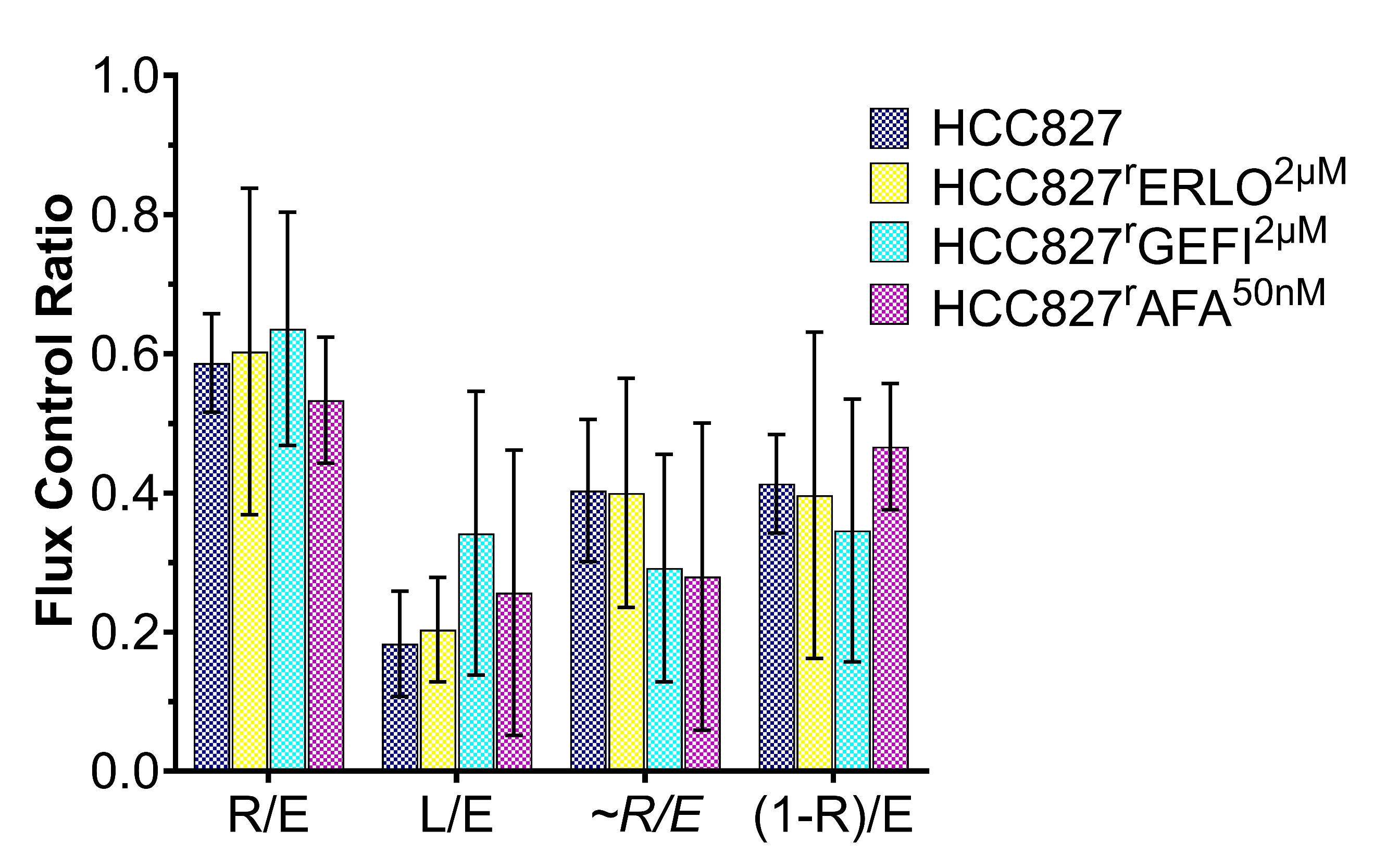
**A1. A2.**


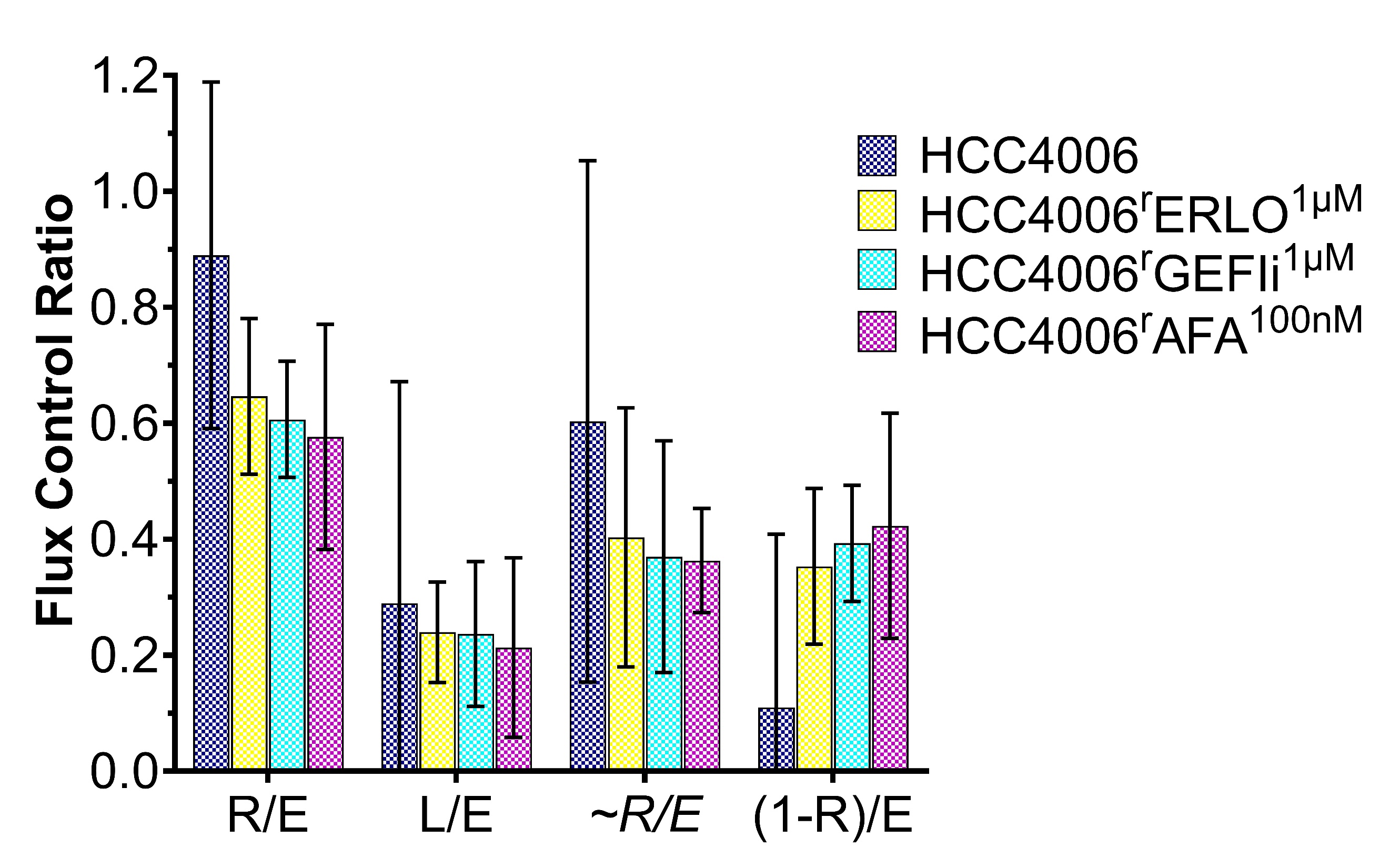

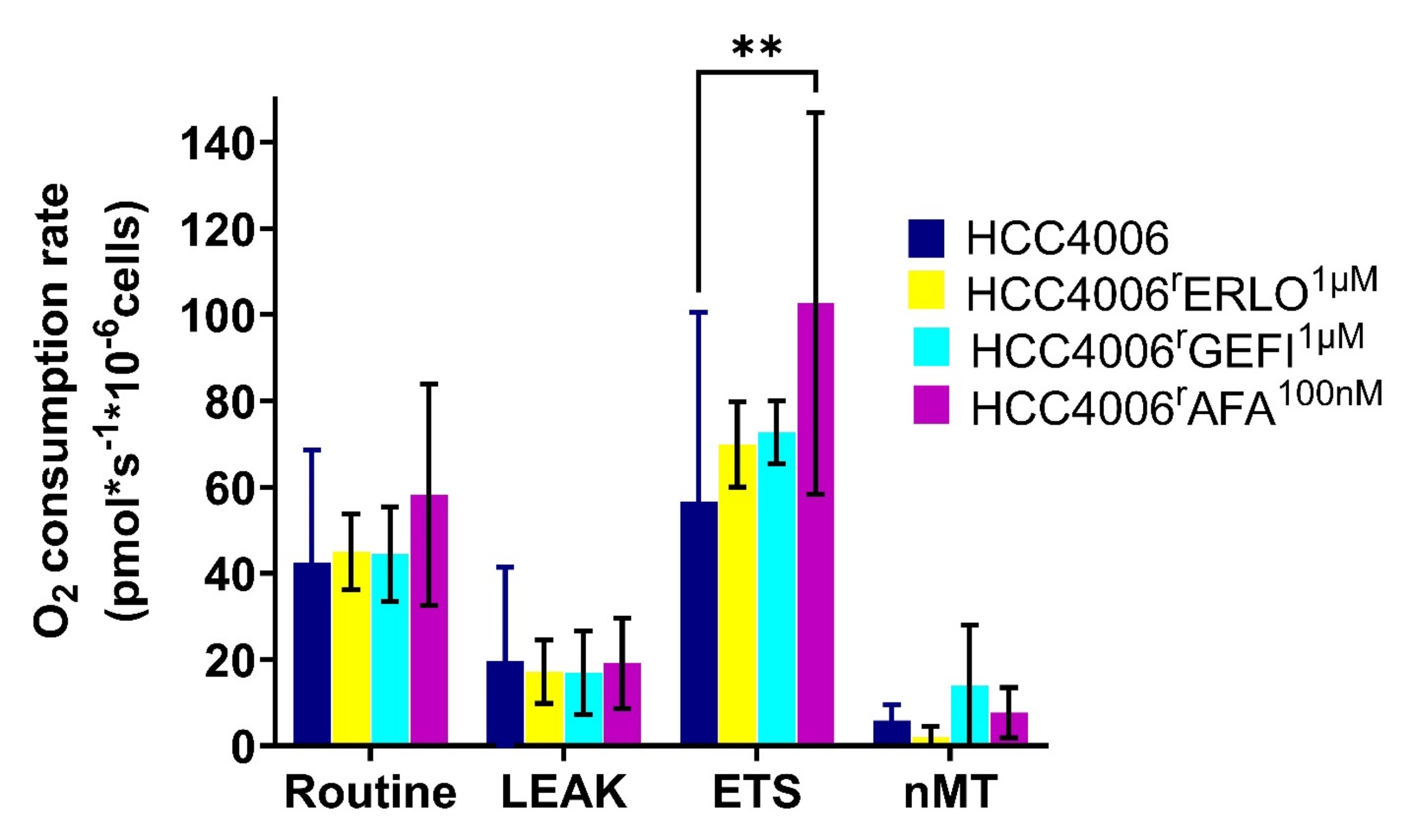
**B1. B2.**

**Supplementary Figure 8. Summary of high-resolution respirometry results of HCC827, HCC4006 andrespective EGFR tyrosine kinase inhibitor-adapted sublines.** Data are presented as oxygen consumption rates and flux control ratios. Bars represent the mean ± S.D. of three independent experiments. Differences were analysed for statistical significance (p<0.05) by student’s t-test. *p<0.05, **p<0.01**. LEAK – reduction in oxygen consumption related to addition of the ATP synthase inhibitor oligomycin, ETS - electron transfer system capacity (addition of FCCP (carbonyl cyanide 4-(trifluoromethoxy)phenylhydrazone) causes mitochondrial membrane permeabilization), resulting in maximum respiration/ oxygen consumption, nMT - non-mitochondrial oxygen consumption (Complex III inhibitor antimycin A addition result in respiration suppression).

**Supplementary Table 1. Doubling times of HCC827 and HCC4006 and their EGFR tyrosine kinase inhibitor-adapted sublines in the absence and presence of drug.** Values represent the mean of three independent biological replicates ± S.D. Statistical significance was tested using two-way ANOVA with post-hoc Tukey’s pairwise comparison test. * p<0.05

|  | Doubling time (Hrs) | | | |
| --- | --- | --- | --- | --- |
|  | HCC827 | HCC827^r^ERLO^2µM^ | HCC827^r^GEFI^2µM^ | HCC827^r^AFA^50nM^ |
| No drug | 12.8 ± 0.8 | 18.6 ± 4.3 | 20.2 ± 7.3 | 15.1 ± 5.6 |
| With drug | - | 23.4 ± 2.8* | 28.8 ± 0.5* | 20.9 ± 4.4 |

|  | Doubling time (Hrs) | | | |
| --- | --- | --- | --- | --- |
|  | HCC4006 | HCC4006^r^ERLO^1µM^ | HCC4006^r^GEFIi^1µM^ | HCC4006^r^AFA^100nM^ |
| No drug | 25.4 ± 3.4 | 28.3 ± 6.4 | 23.2 ± 3.0 | 31.5 ± 7.4 |
| With drug | n/a | 29.7 ± 4.8 | 27.8 ± 11.9 | 35.0 ± 16.4 |

**Supplementary Table 2. Sensitivity of HCC827, HCC4006, and their EGFR tyrosine kinase inhibitor-resistant sublines to EGFR tyrosine kinase inhibitors.** IC_50_ values represent mean of three independent biological repeats ± S.D. Drug response was determined by MTT assay after a 120h incubation period. IC_50_ values were calculated using Calcusyn (Version 1.1, Biosof 1996). IC_50_ - half maximal inhibitory concentrations.

| Cell line | Drug | | | | | | | |
| --- | --- | --- | --- | --- | --- | --- | --- | --- |
|  | **Erlotinib** | | **Gefitinib** | | **Afatinib** | | **Osimertinib** | |
|  | **IC_50_** (µM) | **RF** | **IC_50_** (µM) | **RF** | **IC_50_** (nM) | **RF** | **IC_50_** (µM) | **RF** |
| HCC827 | 0.08±0.02 | n/a | 0.08±0.02 | n/a | 1.32±0.47 | n/a | 0.0004±0.0002 | n/a |
| HCC827^r^ERLO^2µM^ | 22.02±3.19 | 282.6 | 21.80±1.45 | 290.5 | 2232.96±628.34 | 1690.7 | 1.53±0.36 | 3642.9 |
| HCC827^r^GEFI^2µM^ | 32.54±6.28 | 417.6 | 13.36±1.58 | 178.1 | 2359.51±238.97 | 1786.5 | 2.74±0.42 | 6523.8 |
| HCC827^r^AFA^50nM^ | 3.29±1.19 | 42.2 | 13.88±1.50 | 185.0 | 2417.76±262.63 | 1830.6 | 1.14±0.31 | 2714.3 |

| Cell line | Drug | | | | | | | |
| --- | --- | --- | --- | --- | --- | --- | --- | --- |
|  | **Erlotinib** | | **Gefitinib** | | **Afatinib** | | **Osimertinib** | |
|  | **IC_50_** (µM) | **RF** | **IC_50_** (µM) | **RF** | **IC50** (nM) | **RF** | **IC_50_** (µM) | **RF** |
| HCC4006 | 0.03±0.01 | n/a | 0.03±0.00 | n/a | 0.15±0.05 | n/a | 0.004±0.003 | n/a |
| HCC4006^r^ERLO^1µM^ | 13.65±4.19 | 519.7 | 13.86±1.59 | 473.1 | 1715.55±308.09 | 11319.0 | 4.01±1.35 | 1000.8 |
| HCC4006^r^GEFI^1µM^ | 20.20±2.52 | 769.3 | 33.38±14.03 | 1139.9 | 3761.06±883.09 | 24815.1 | 2.62±0.49 | 653.9 |
| HCC4006^r^AFA^100nM^ | 11.18±3.98 | 425.9 | 13.33±0.14 | 455.0 | 2533.50±664.04 | 16715.8 | 2.60±0.25 | 650.0 |

**Supplementary Table 3. Sensitivity of HCC827, HCC4006, and their EGFR tyrosine kinase inhibitor-resistant sublines to cytotoxic anti-cancer drugs.** IC_50_ values represent mean of three independent biological repeats ± S.D. Drug response was determined by MTT assay after a 120h incubation period. IC_50_ values were calculated using Calcusyn (Version 1.1, Biosof 1996). IC_50_ - half maximal inhibitory concentrations.

| Cell line | Drug | | | | | |
| --- | --- | --- | --- | --- | --- | --- |
|  | **Cisplatin** | | **Paclitaxel** | | **Vincristine** | |
|  | **IC_50_** (ng/mL) | **RF** | **IC_50_** (nM) | **RF** | **IC_50_** (nM) | **RF** |
| HCC827 | 2353.34±235.15 | n/a | 0.90±0.11 | n/a | 18.58±9.47 | n/a |
| HCC827^r^ERLO^2µM^ | 3166.98±596.87 | 1.35 | 5.58±0.91 | 6.20 | 2.96±0.40 | 0.16 |
| HCC827^r^GEFI^2µM^ | 1430.77±531.86 | 0.61 | 6.74±0.22 | 7.49 | 6.19±0.84 | 0.33 |
| HCC827^r^AFA^50nM^ | 1617.92±662.61 | 0.69 | 3.04±1.44 | 3.38 | 126.78±53.01 | 8.04 |

| Cell line | Drug | | | | | |
| --- | --- | --- | --- | --- | --- | --- |
|  | **Cisplatin** | | **Paclitaxel** | | **Vincristine** | |
|  | **IC_50_** (ng/mL) | **RF** | **IC_50_** (nM) | **RF** | **IC_50_** (nM) | **RF** |
| HCC4006 | 1477.36±540.32 | n/a | 1.14±0.20 | n/a | 0.84±0.21 | n/a |
| HCC4006^r^ERLO^1µM^ | 4819.52±853.62 | 3.26 | 24.71±4.18 | 21.68 | 14.83±9.16 | 17.55 |
| HCC4006^r^GEFI^1µM^ | 6768.33±854.69 | 4.58 | 1.09±0.03 | 0.96 | 1.74±0.67 | 2.06 |
| HCC4006^r^AFA^100nM^ | 5082.04±342.74 | 3.44 | 70.06±19.69 | 61.46 | 4604±120 | 5451 |

**Supplementary Table 4. Sensitivity of HCC827, HCC4006, and their EGFR tyrosine kinase inhibitor-resistant sublines to different kinase inhibitors.** Drug response was determined by MTT assay after a 120h incubation period. IC_50_ values were calculated using Calcusyn (Version 1.1, Biosof 1996). Resistance factors (RF) were calculated as follows IC_50_ drug-resistant subline/ IC_50_ respective parental cell line. Values represent mean of three independent biological repeats ± S.D. IC_50_ - half maximal inhibitory concentrations

| Cell line | Drug | | | | | | | | | |
| --- | --- | --- | --- | --- | --- | --- | --- | --- | --- | --- |
|  | Cabozantinib | | Trametinib | | Alpelisib | | LY294002 | | AT13148 | |
|  | **IC_50_** (µM) | **RF** | **IC_50_** (µM) | **RF** | **IC_50_** (µM) | **RF** | **IC_50_** (µM) | **RF** | **IC_50_**(µM) | **RF** |
| HCC827 | 33.99±9.19 | n/a | 25.56±2.88 | n/a | 28.73±0.98 | n/a | 26.10±1.40 | n/a | 43.89 | n/a |
| HCC827^r^ERLO^2µM^ | 0.062±0.032 | 0.0018 | 0.035 ±0.003 | 0.0014 | 9.45±2.43 | 0.33 | 13.54±0.82 | 0.52 | 44.64 | 1.02 |
| HCC827^r^GEFI^2µM^ | 0.009±0.003 | 0.0004 | 0.061±0.034 | 0.0024 | 6.82±0.67 | 0.24 | 7.79±1.75 | 0.30 | 3.71 | 0.08 |
| HCC827^r^AFA^50nM^ | 6.03±0.53 | 0.18 | 29.486±4.18 | 1.15 | 36.30±6.16 | 1.26 | 19.72±2.89 | 0.76 | 35.30 | 0.80 |

| Cell line | Drug | | | | | | | | | |
| --- | --- | --- | --- | --- | --- | --- | --- | --- | --- | --- |
|  | Cabozantinib | | Trametinib | | Alpelisib | | LY294002 | | AT13148 | |
|  | **IC_50_** (µM) | **RF** | **IC_50_** (µM) | **RF** | **IC_50_** (µM) | **RF** | **IC_50_** (µM) | **RF** | **IC_50_**(µM) | **RF** |
| HCC4006 | 8.52±1.44 | n/a | 0.017±0.005 | n/a | 2.84±0.46 | n/a | 12.61±3.00 | n/a | 2.30 | n/a |
| HCC4006^r^ERLO^1µM^ | 6.58±0.80 | 0.77 | 0.105±0.004 | 6.30 | 7.51±1.93 | 2.64 | 8.41±0.62 | 0.67 | 6.67 | 2.90 |
| HCC4006^r^GEFI^1µM^ | 8.73±1.62 | 1.02 | 0.043±0.007 | 2.58 | 8.27±1.44 | 2.91 | 13.03±2.88 | 1.03 | 15.24 | 6.63 |
| HCC4006^r^AFA^100nM^ | 2.48±0.64 | 0.29 | 0.073±0.010 | 4.36 | 4.15±1.14 | 1.46 | 3.65±0.53 | 0.29 | 11.34 | 4.94 |

**Supplementary Table 5. Determination of the IC_25_ and IC_50_ values for different kinase inhibitors in HCC827, HCC4006, and their EGFR tyrosine kinase inhibitor-resistant sublines to different kinase inhibitors.** Drug response was determined by MTT assay after a 120h incubation period. IC_50_ values were calculated using Calcusyn (Version 1.1, Biosof 1996). IC_50_ values represent mean of three independent biological repeats ± S.D. IC_50_ - half maximal inhibitory concentrations, IC_25_ - concentration that inhibit cell viability by 25%.

| Cell line | Drug | | | | | | | |
| --- | --- | --- | --- | --- | --- | --- | --- | --- |
|  | Cabozantinib (μM) | | Trametinib (μM) | | Alpelisib (μM) | | LY294002 (μM) | |
|  | **IC_25_** | **IC_50_** | **IC_25_** | **IC_50_** | **IC_25_** | **IC_50_** | **IC_25_** | **IC_50_** |
| HCC827 | **0.037** | **0.062** | **0.009** | **0.035** | **4.188** | **9.454** | **7.419** | **13.539** |
| HCC827^r^ERLO^2µM^ | **0.003** | **0.009** | **0.010** | **0.061** | **3.895** | **6.815** | **3.787** | **7.791** |
| HCC827^r^GEFI^2µM^ | **3.043** | **6.026** | **11.304** | **29.486** | **23.508** | **36.303** | **11.013** | **19.720** |
| HCC4006 | **3.226** | **6.580** | **0.015** | **0.105** | **1.463** | **7.511** | **1.235** | **8.405** |
| HCC4006^r^ERLO^1µM^ | **2.963** | **8.728** | **0.012** | **0.043** | **1.413** | **8.269** | **4.453** | **13.029** |
| HCC4006^r^GEFI^1µM^ | **0.018** | **2.479** | **0.022** | **0.073** | **0.755** | **4.148** | **1.000** | **3.648** |

**Supplementary Table 6: Impact of different kinase inhibitors on the sensitivity of EGFR tyrosine kinase-adapted HCC827 and HCC4006 sublines to their respective drugs of adaptation.** Drug response was determined by MTT assay after a 120h incubation period. IC_50_ values were calculated using Calcusyn (Version 1.1, Biosof 1996). IC_50_ values represent mean of three independent biological repeats (n=4 for IC_25_ of alpelisib and LY294002 when treated along with erlotinib, n=5 for trametinib when treated along with IC_25_ of trametinib) ± S.D. Resistance factors (RF) were calculated as follows IC_50_ drug-resistant subline/ IC_50_ respective parental cell line. IC_50_ - half maximal inhibitory concentrations, IC_25_ - concentration that inhibit cell viability by 25%.

| Cell line | Untreated | | Drug (IC_25_ treated) | | | | | | | |
| --- | --- | --- | --- | --- | --- | --- | --- | --- | --- | --- |
|  |  |  | Cabozantinib | | Trametinib | | Alpelisib | | LY294002 | |
|  | **IC_50_** (µM) | **RF** | **IC_50_** (µM) | **RF** | **IC_50_** (µM) | **RF** | **IC_50_** (µM) | **RF** | **IC_50_** (µM) | **RF** |
| HCC827 | 20.77±6.21 | 266.6 | 0.17 ± 0.06 | 2.2 | 3.11 ± 1.32 | 39.9 | 6.24± 0.73 | 80.1 | 5.97±1.52 | 76.6 |
| HCC827^r^ERLO^2µM^ | 8.16± 1.33 | 108.8 | 5.24 ± 2.48 | 69.8 | 6.09 ± 2.53 | 81.2 | 2.63± 0.91 | 35.0 | 5.10±2.17 | 68.0 |
| HCC827^r^GEFI^2µM^ | 1.74± 0.16 | 1319.5 | 0.44 ± 0.25 | 335.8 | 0.37± 0.15 | 282.1 | 0.0014±0.0008 | 1.0 | 0.003±0.0019 | 2.5 |
| HCC4006 | 18.17±1.84 | 691.9 | 35.59± 8.81 | 1355.4 | 29.88±8.77 | 1138.0 | 27.05 ± 18.39 | 1030.3 | 21.94 ± 13.05 | 835.5 |
| HCC4006^r^ERLO^1µM^ | 45.97±4.14 | 1569.5 | 6.68 ± 1.91 | 228.1 | 47.77±9.34 | 1631.0 | 16.06 ± 2.45 | 548.4 | 51.58 ± 11.13 | 1761.1 |
| HCC4006^r^GEFI^1µM^ | 2.24± 1.27 | 14807.9 | 3.28 ± 0.55 | 21645.7 | 2.81±1.67 | 18529.5 | 3.10 ± 0.73 | 20468.4 | 4.08 ± 1.74 | 26886.9 |
| Cell line | **Untreated** | | **Drug (IC_50_ treated)** | | | | | | | |
|  |  |  | Cabozantinib | | Trametinib | | Alpelisib | | LY294002 | |
|  | **IC_50_** (µM) | **RF** | **IC_50_** (µM) | **RF** | **IC_50_** (µM) | **RF** | **IC_50_** (µM) | **RF** | **IC_50_** (µM) | **RF** |
| HCC827 | 20.77±6.21 | 266.5 | 0.016± .008 | 0.2 | 2.29± 1.54 | 29.3 | 23.29 ± 4.44 | 298.9 | 22.97 ± 4.80 | 294.8 |
| HCC827^r^ERLO^2µM^ | 8.16 ± 1.33 | 108.8 | 5.80 ± 1.79 | 77.3 | 3.44± 0.56 | 45.8 | 1.83 ± 0.34 | 24.4 | 4.51 ± 2.20 | 60.1 |
| HCC827^r^GEFI^2µM^ | 1.74 ± 0.16 | 1319.5 | 0.03 ± 0.02 | 20.1 | 0.14± 0.12 | 104.9 | 0.0008±0.0007 | 0.6 | 0.026 ± 0.007 | 19.4 |
| HCC4006 | 18.17±1.84 | 691.9 | 30.07± 9.56 | 1145.3 | 52.06±23.72 | 1982.5 | 23.70 ± 0.62 | 902.7 | 36.78 ± 3.74 | 1400.8 |
| HCC4006^r^ERLO^1µM^ | 45.97±4.14 | 1569.5 | 2.31 ± 1.42 | 78.9 | 46.34± 3.04 | 1582.4 | 21.61 ± 5.36 | 737.8 | 23.97 ± 1.13 | 818.5 |
| HCC4006^r^GEFI^1µM^ | 2.24 ± 1.27 | 14807.9 | 1.22 ± 0.78 | 8065.6 | 2.19± 2.16 | 14418.0 | 3.57 ± 0.93 | 23555.4 | 3.40 ± 0.67 | 22455.5 |

**Supplementary Table 7. HDAC inhibitor concentrations inhibit HDACs with some level of specificity.**

| **HDAC inhibitor** | **Concentration** | **HDACs inhibited** | **Reference** |
| --- | --- | --- | --- |
| Apicidin | 200nM | HDAC (1)/2/3 | Bantscheff et al., 2011 |
| Bufexamac | 30µM | HDAC 6/10 | Bantscheff et al., 2011 |
| CI-994 | 2µM | HDAC 1/2/3/(8) | Bantscheff et al., 2011 |
| Compound 2 | 40µM | HDAC 8 | Krennhrubec et al., 2007  Oehme et al., 2009 |
| Fimepinostat/ CUDC-994 | 5nM | HDAC 1/2/3/10 | <https://www.selleckchem.com/products/pi3k-hdac-inhibitor-i.html> |
| Droxinostat | 15µM | HDAC 6/8 | <https://www.selleckchem.com/products/Droxinostat.html> |
| Mocetinostat | 300nM | HDAC 1/(2)/(3)/(11) | <https://www.selleckchem.com/products/MGCD0103(Mocetinostat).html> |
| Entinostat/ MS-275 | 250nM | HDAC 1/2/3 | Deubzer et al., 2008  Witt et al., 2009  Mustafa & Krämer, 2023 |
| Panobinostat | 10nM | pan | Witt et al., 2009 |
| PCI-24781 | 500nM | pan | Bantscheff et al., 2011 |
| PCI-34051 | 4µM | HDAC 8 | Balasubramanian et al., 2008  Bantscheff et al., 2011 |
| Romidepsin/ FK-228* | 40nM | HDAC 1/2/8 | Bantscheff et al., 2011  Mustafa & Krämer, 2023 |
| Trichostatin A | 75nM | pan | Bantscheff et al., 2011 |
| Tubacin | 2.5µM | HDAC 6 | Witt et al., 2009 |
| Tubastatin A | 7.5µM | HDAC 6/10 | Géraldy et al., 2019 |
| Vorinostat/ SAHA* | 1µM | pan | Bantscheff et al., 2011 |

* Approved for the treatment of cutaneous T-cell lymphoma (CTCL) and peripheral T-cell lymphoma (Mustafa & Krämer, 2023)

*References*

Balasubramanian S, Ramos J, Luo W, Sirisawad M, Verner E, Buggy JJ. A novel histone deacetylase 8 (HDAC8)-specific inhibitor PCI-34051 induces apoptosis in T-cell lymphomas. Leukemia. 2008 May;22(5):1026-34. doi: 10.1038/leu.2008.9.

Bantscheff M, Hopf C, Savitski MM, Dittmann A, Grandi P, Michon AM, Schlegl J, Abraham Y, Becher I, Bergamini G, Boesche M, Delling M, Dümpelfeld B, Eberhard D, Huthmacher C, Mathieson T, Poeckel D, Reader V, Strunk K, Sweetman G, Kruse U, Neubauer G, Ramsden NG, Drewes G. Chemoproteomics profiling of HDAC inhibitors reveals selective targeting of HDAC complexes. Nat Biotechnol. 2011 Mar;29(3):255-65. doi: 10.1038/nbt.1759.

Géraldy M, Morgen M, Sehr P, Steimbach RR, Moi D, Ridinger J, Oehme I, Witt O, Malz M, Nogueira MS, Koch O, Gunkel N, Miller AK. Selective Inhibition of Histone Deacetylase 10: Hydrogen Bonding to the Gatekeeper Residue is Implicated. J Med Chem. 2019 May 9;62(9):4426-4443. doi: 10.1021/acs.jmedchem.8b01936.

Krennhrubec K, Marshall BL, Hedglin M, Verdin E, Ulrich SM. Design and evaluation of 'Linkerless' hydroxamic acids as selective HDAC8 inhibitors. Bioorg Med Chem Lett. 2007 May 15;17(10):2874-8. doi: 10.1016/j.bmcl.2007.02.064.

Mustafa AM, Krämer OH. Pharmacological Modulation of the Crosstalk between Aberrant Janus Kinase Signaling and Epigenetic Modifiers of the Histone Deacetylase Family to Treat Cancer. Pharmacol Rev. 2023 Jan;75(1):35-61. doi: 10.1124/pharmrev.122.000612.

Oehme I, Deubzer HE, Wegener D, Pickert D, Linke JP, Hero B, Kopp-Schneider A, Westermann F, Ulrich SM, von Deimling A, Fischer M, Witt O. Histone deacetylase 8 in neuroblastoma tumorigenesis. Clin Cancer Res. 2009 Jan 1;15(1):91-9. doi: 10.1158/1078-0432.CCR-08-0684.

Witt O, Deubzer HE, Milde T, Oehme I. HDAC family: What are the cancer relevant targets? Cancer Lett. 2009 May 8;277(1):8-21. doi: 10.1016/j.canlet.2008.08.016.

**Supplementary Table 8. Effect of HDAC inhibitors on the viability of the project cell lines.** Cell viability was determined by MTT assay after a 120h incubation period. The data are from three independent biological repeats, mean ± S.D.

| **HDAC inhibitor** | **Cell viability (%)** | | | | | | | | |
| --- | --- | --- | --- | --- | --- | --- | --- | --- | --- |
|  | **HCC827** | | | | **HCC4006** | | | | |
|  | **HCC827** | **^r^ERLO^2µM^** | **^r^GEFI^2µM^** | **^r^AFA^50nM^** | **HCC4006** | **^r^ERLO^1µM^** | **^r^GEFI^1µM^** | **^r^AFA^100nM^** |  |
| **Abexinostat** | 76% ± 17 | 44% ± 17 | 68% ± 17 | 71% ± 7 | 44% ± 5 | 48% ± 10 | 65% ± 28 | 40% ± 15 |  |
| **Panobinostat** | 78% ± 7 | 79% ± 8 | 97% ± 3 | 55% ± 12 | 56% ± 5 | 62% ± 8 | 77% ± 24 | 64% ± 24 |  |
| **TSA** | 82% ± 10 | 90% ± 7 | 91% ± 6 | 63% ± 16 | 72% ± 11 | 71% ± 7 | 75% ± 14 | 71% ± 21 |  |
| **Vorinostat/**  **SAHA** | 89% ± 10 | 56% ± 6 | 75% ± 12 | 66% ± 4 | 43% ± 2 | 47% ± 18 | 60% ± 23 | 47% ± 20 |  |
| **Apicidin** | 61% ± 13 | 28% ± 7 | 9% ± 11 | 70% ± 30 | 25% ± 13 | 41% ± 17 | 54% ± 24 | 36% ± 13 |  |
| **CI-994** | 82% ± 10 | 73% ± 5 | 88% ± 10 | 55% ± 11 | 39% ± 28 | 49% ± 43 | 52% ± 49 | 49% ± 29 |  |
| **CUDC -994** | 97% ± 4 | 85% ± 10 | 74% ± 27 | 64% ± 11 | 53% ± 14 | 58% ± 17 | 72% ± 34 | 61% ± 18 |  |
| **Entinostat/**  **MS-275** | 99% ± 1 | 71% ± 8 | 87% ± 12 | 66% ± 6 | 55% ± 26 | 68% ± 32 | 71% ± 29 | 68% ± 23 |  |
| **Mocetinostat** | 98% ± 4 | 49% ± 6 | 61% ± 16 | 62% ± 4 | 51% ± 9 | 59% ± 6 | 72% ± 24 | 59% ± 18 |  |
| **Tubacin** | 100% ± 0 | 91% ± 4 | 67% ± 14 | 74% ± 9 | 60% ± 12 | 71% ± 15 | 68% ± 6 | 64% ± 18 |  |
| **Bufexamac** | 92% ± 5 | 71% ± 11 | 80% ± 17 | 77% ± 11 | 32% ± 25 | 36% ± 33 | 48% ± 46 | 42% ± 25 |  |
| **Tubastatin A** | 92% ± 9 | 81% ± 3 | 89% ± 7 | 63% ± 17 | 66% ± 10 | 76% ± 17 | 85% ± 18 | 72% ± 21 |  |
| **Droxinostat** | 85% ± 5 | 62% ± 7 | 57% ± 18 | 62% ± 9 | 26% ± 19 | 30% ± 27 | 32% ± 29 | 25% ± 16 |  |
| **Cpd 2** | 87% ± 11 | 72% ± 4 | 65% ± 17 | 63% ± 14 | 33% ± 22 | 50% ± 16 | 56% ± 36 | 50% ± 19 |  |
| **PCI-34051** | 81% ± 8 | 78% ± 12 | 70% ± 18 | 74% ± 11 | 62% ± 6 | 63% ± 7 | 73% ± 13 | 64% ± 20 |  |
